# Epistasis at the SARS-CoV-2 RBD Interface and the Propitiously Boring Implications for Vaccine Escape

**DOI:** 10.1101/2021.08.30.458225

**Published:** 2021-08-30

**Authors:** Nash D. Rochman, Guilhem Faure, Yuri I. Wolf, Peter L. Freddolino, Feng Zhang, Eugene V. Koonin

**Affiliations:** 1 National Center for Biotechnology Information, National Library of Medicine, Bethesda, MD 20894; 2 Broad Institute of MIT and Harvard, Cambridge, MA 02142;; 3 Department of Biological Chemistry, University of Michigan Medical School, Ann Arbor, MI, USA;; 4 Department of Computational Medicine and Bioinformatics, University of Michigan Medical School, Ann Arbor, MI, USA;; 5 Howard Hughes Medical Institute, Massachusetts Institute of Technology, Cambridge, MA 02139;; 6 McGovern Institute for Brain Research, Massachusetts Institute of Technology, Cambridge, MA 02139;; 7 Department of Brain and Cognitive Sciences, Massachusetts Institute of Technology, Cambridge, MA 02139;; 8 Department of Biological Engineering, Massachusetts Institute of Technology, Cambridge, MA 02139

**Keywords:** SARS-CoV-2, Delta Variant, Gamma Variant, Escape Mutants, Epistasis, Protein Structure Modelling, Rosetta

## Abstract

At the time of this writing, August 2021, potential emergence of vaccine escape variants of severe acute respiratory syndrome coronavirus 2 (SARS-CoV-2) is a grave global concern. The interface between the receptor-binding domain (RBD) of SARS-CoV-2 spike (S) protein and the host receptor (ACE2) overlap with the binding site of principal neutralizing antibodies (NAb), limiting the repertoire of viable mutations. Nonetheless, variants with multiple mutations in the RBD have rose to dominance. Non-additive, epistatic relationships among RBD mutations are apparent, and assessing the impact of such epistasis on the mutational landscape is crucial. Epistasis can substantially increase the risk of vaccine escape and cannot be completely characterized through the study of the wild type (WT) alone. We employed protein structure modeling using Rosetta to compare the effects of all single mutants at the RBD-NAb and RBD-ACE2 interfaces for the WT, Gamma (417T, 484K, 501Y), and Delta variants (452R, 478K). Overall, epistasis at the RBD surface appears to be limited and the effects of most multiple mutations are additive. Epistasis at the Delta variant interface weakly stabilizes NAb interaction relative to ACE2, whereas in the Gamma variant, epistasis more substantially destabilizes NAb interaction. These results suggest that the repertoire of potential escape mutations for the Delta variant is not substantially different from that of the WT, whereas Gamma poses a moderately greater risk for enhanced vaccine escape. Thus, the modest ensemble of mutations relative to the WT shown to reduce vaccine efficacy might constitute the majority of all possible escape mutations.

## Introduction

When severe acute respiratory syndrome coronavirus 2 (SARS-CoV-2), first emerged as a global public health concern early in 2020, there has been considerable debate regarding whether the low mutation rate of the virus and the relatively inflexible receptor-binding domain of the antigenic spike (S) protein would admit robust host adaptation(1, 2). By 2021, it became clear that SARS-CoV-2 has access to a broad mutational repertoire enabling extensive diversification(3) and that without vaccination, SARS-CoV-2 would likely result in substantial global disease burden for a protracted period(4, 5). The development of multiple, effective vaccines against SARS-CoV-2(6) make it possible to dramatically reduce this burden. However, at the time of writing, in August, 2021, the majority of the global population remains unvaccinated and the potential emergence of vaccine escape variants(7) is of major international concern https://www.cdc.gov/coronavirus/2019-ncov/variants/variant-info.html.

The interface between the receptor-binding domain (RBD) of the S protein and the host receptor (ACE2) largely overlaps with the binding sites for the most potent neutralizing antibodies (NAb)(8, 9), limiting the scope of viable mutations. Nevertheless, multiple variants containing single mutations in the RBD that, to different extents, reduce NAb binding have begun to circulate(8–10). Moreover, variants with multiple mutations in the RBD have risen to dominance outcompeting the wild type (WT, identical to Wuhan-Hu-1) and single mutants (see below). This dynamic of SARS-CoV-2 variants could result from non-additive, epistatic, interactions among the mutated sites(10, 11) or simply from additive effects of multiple mutations(11). The effects of all single mutations in the RBD relative to the WT have been studied(8, 12). Epistasis among RBD mutations has the potential to substantially increase the risk of escape variant emergence and cannot be characterized through the study of the WT alone.

Using the Rosetta software suite https://rosettacommons.org(13), we set out to study and compare the effects of all single non-synonymous mutants at the RBD-NAb and RBD-ACE2 interfaces for the WT, Gamma variant (417T, 484K, 501Y), and Delta variant (452R, 478K). The Gamma and Delta variants were considered to be of the greatest relevance at the time of writing because both, particularly Delta, have become dominant in different regions of the world, and both were still on the ascent(14, 15). We establish the distribution of RBD mutations on the plane bounded by the costs of ACE2 and NAb binding and classify the direction and magnitude of epistatic interactions between variant mutations and the broader mutational repertoire. The results reveal only weak epistasis, which is more pronounced for the Gamma than for the Delta variant, and accordingly, a limited potential for the emergence of escape variants.

## Results

### Rationale

**Figure 1.**
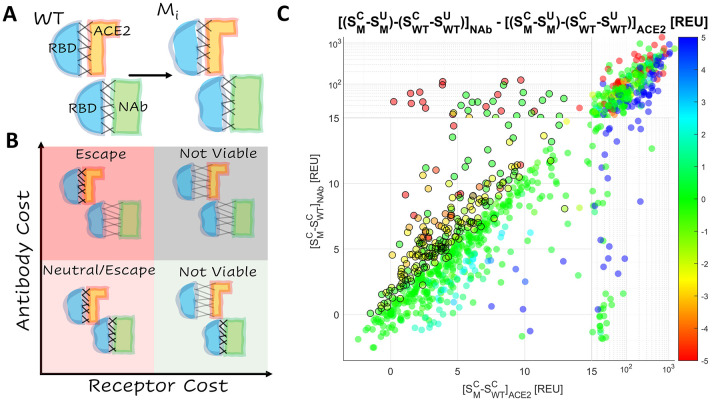
Landscape of Vaccine Escape Mutants for the WT RBD **A.** Cartoon depicting unique conformational changes to the RBD (blue) in complex with ACE2 (orange) and the NAb (green) associated with the same mutation. **B.** Cartoon depicting the landscape of vaccine escape mutations (the plane of receptor cost vs antibody cost). **C.** Landscape of vaccine escape mutations for the WT RBD. Circles with a black outline are NAb escape candidates. Color indicates propensity for escape as measured by *ΔΔG*.

Epistatic interactions among mutations in the RBD are of interest and concern because they might substantially increase the risk of vaccine escape. Mutations in the RBD subtly change the shapes of the interfaces between RBD and ACE2, and between RBD and NAb (Figure 1A). While the structure of the WT RBD-ACE2 interface is highly similar to that of the RBD-NAb interface (see below), a single mutation in the RBD can result in distinct shape changes in both interfaces. These changes can be depicted by the position of each mutant on the plane bounded by the receptor binding cost and the antibody cost (Figure 1B). The cost is the increase (positive cost) or decrease (negative cost) in the ACE2 or Nab binding affinity relative to the WT. The four quadrants of this plane represent four broad categories of mutations. Mutations in the top, right quadrant are strongly destabilizing relative to both ACE2 and the NAb. The bottom, right quadrant contains mutants that strongly destabilize the interaction with ACE2 but not with NAb. Most mutants in these quadrants are not evolutionarily viable. Mutants in the bottom, left quadrant stabilize or only weakly destabilize both interfaces. These mutations may or may not provide a selective advantage to the virus depending on the fraction of the host population that has been vaccinated or has recovered from prior infection. The top, left quadrant contains mutations most likely to admit vaccine escape, those which strongly destabilize the interaction with NAb but not with ACE2.

### Single-mutant vaccine escape candidates for the wild type RBD

Starting with the two crystal structures of interest, RBD in complex with ACE2 https://www.rcsb.org/structure/6M0J(16) and RBD in complex with the NAb CV30, https://www.rcsb.org/structure/6XE1, we generated a representative ensemble of 50 native conformations per complex following standard Rosetta protocols (see Methods and Discussion for details). Although NAb that bind epitopes, which do not overlap with the RBD have been identified(8), at the time of writing, the antibodies most critical for assessing the risk of vaccine escape appear to reside within the RBD(9) and are well represented by CV30. Regions important for antibody binding are known to overlap broadly among human coronaviruses(17). We then identified the RBD residues at the interface for each conformation and, in all conformations, introduced all single amino acid substitutions at these sites. For the WT and Delta variant, 52 residues (Table 1) were identified at the interface of at least one conformation for either complex. For the Gamma variant, 4 additional residues were identified. Sites 480 and 488 are connected by a disulfide bond and were found to be unsuitable for substitution.

All 19 substitutions in each of the remaining 54 sites were investigated, with the exception of WT reversion for the variants. Altogether, this analysis produced 307,300 structures, which necessitated the development of a computationally efficient protocol. To meet this need, mutants were introduced into each conformation without repacking of adjacent sidechains or backbone minimization. This minimalist approach yielded favorable comparisons to available experimental data (see below). However, generally, substitutions might introduce steric or charge clashes within the conformations, in which the mutations were introduced (without repacking and minimization). Inference of the relative change in binding affinity for the ACE2 and NAb complexes is limited for such mutations. However, we observed a favorable comparison to experimental data in this respect as well, whereby few experimentally predicted escape mutations (relative to the WT) fall into this, inference-limited, category (see below).

Structure stability was estimated by the total score, *S*, in arbitrary units produced by the empirically-driven Rosetta Energy Function 2015(18) (labelled REU for “Rosetta Energy Units”, https://new.rosettacommons.org/docs/latest/rosetta_basics/Units-in-Rosetta). The total score was calculated for each of the 50 conformations of the NAb and ACE2 complexes generated, and the mean value was assessed with, $S_{M}^{C}$, and without, $S_{WT}^{C}$, the mutation. The receptor cost and antibody cost were estimated as ${\left[S_{M}^{C}-S_{WT}^{C}\right]}_{ACE2}$ and ${\left[S_{M}^{C}-S_{WT}^{C}\right]}_{NAb}$, respectively. The interface free energy (*ΔG*) was also more directly approximated by the difference between the total score of the unbound state and the complex, *S*^*C*^ − *S*^*U*^. The effect of the mutation on this value (*ΔΔG*) was reported for both complexes, $\left(S_{M}^{C}-S_{M}^{U}\right)-\left(S_{WT}^{C}-S_{WT}^{U}\right)$. Figure 1C shows the distribution of RBD mutations on the plane bounded by the ACE2 and NAb binding costs and putative NAb escape candidates, for which ${\left[S_{M}^{C}-S_{WT}^{C}\right]}_{NAb}-{\left[S_{M}^{C}-S_{WT}^{C}\right]}_{ACE2}>1$ or *ΔΔG*_*NAb*_−*ΔΔG*_*ACE2*_*>1* and ${\left[S_{M}^{C}-S_{WT}^{C}\right]}_{ACE2}<13$. The threshold value of 13 was selected to remove from consideration mutations that likely produce steric or charge clashes in the structure; few experimentally validated escape candidates were observed above this value (see below).

Mutants showed strong clustering along the diagonal (identity line: receptor cost is equal to antibody cost), indicating that most mutations similarly affected the WT RBD-ACE2 and RBD-NAb complexes. Mutations in the top, left quadrant of the plane, which corresponds to strong destabilization of the interaction with NAb but not with ACE2, are the strongest candidates for vaccine escape, followed by those in the bottom, left quadrant, which includes weakly destabilizing mutations. The selective advantage (or lack thereof) of mutations in this quadrant depends on the fraction of the host population that has been vaccinated or has recovered from prior infection (see also Discussion). In a fully vaccinated population, mutations that substantially reduce infectivity through the destabilization of receptor binding could still provide a selective advantage. In particular, multiple mutations in 6 sites (417, 477, 484, 491, 493, 499) were found to substantially destabilize the RBD-NAb complex relative to the RBD-ACE2 complex (Figure S1). Additionally, we identified site 453 to harbor mutations that simultaneously stabilize the RBD-ACE2 complex and destabilize the RBD-NAb complex. These observations are broadly consistent with the results of deep mutational scanning(8, 9, 19–22). Therefore, we conservatively considered all mutations, for which the antibody cost exceeded the receptor cost, to be viable escape candidates.

### Single-mutant vaccine escape candidates for the Gamma and Delta variant RBDs and predicted epistatic interactions

**Figure 2.**
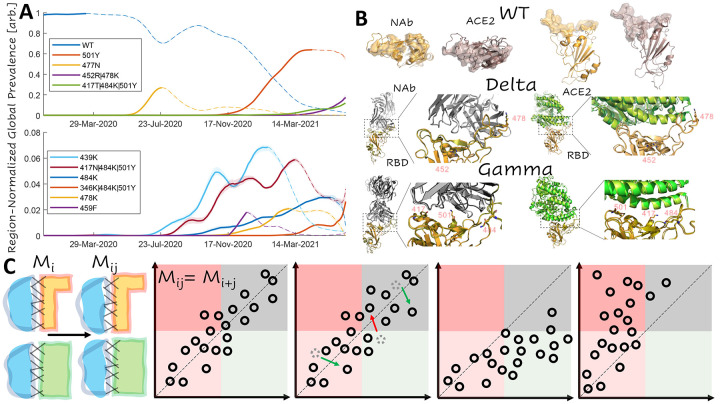
Dominant Trends in Circulating RBD Mutations **A.** Region-normalized global prevalence of the top 10 most common combinations of RBD mutations over time. Lines are solid up to peak prevalence and dashed afterwards. Shading indicates confidence intervals. **B.** Structural comparison of the complexes of ACE2 and NAb with RBD for WT, Delta, and Gamma variants. *Top*: WT footprints including the residues with an interaction within 4A of the partner. *Middle:* Visualization of the Delta variant interface. *Bottom:* Visualization of the Gamma variant interface. Mutations are labeled and represented as sticks. WT structures are superimposed for the RBD of each variant: WT, orange; variant, olive **C.** Cartoon illustrating additive and non-additive (epistatic) interactions between mutations. From left to right: additive, escape-neutral, escape-minimizing, and escape-exacerbating. *Mi, Mj, Mij* denote the effects of the single and double mutants.

Having charted the WT RBD landscape, we sought to identify the most prominent combinations of RBD mutations circulating over the course of the pandemic. As of June, 16^th^, 2021, there were 53 countries, from which more than 1,000 SARS-CoV-2 isolates were contributed to the GISAID(23) database (see Data Availability). For each of these locations, we randomly selected 1,000 isolates and reported the frequency of each combination of RBD mutations among the 53,000 selected isolates over time. Figure 2A displays this region-normalized global prevalence of the 10 most common combinations of RBD mutations. The 6 RBD single-mutants (501Y/Alpha Variant, 477N, 439K, 484K, 478K, and 459F) began emerging between July and November 2020. All these single mutants were eventually displaced by 4 RBD multi-mutants (452R|478K/Delta Variant, 417T|484K|501Y/Gamma Variant, 417N|484K|501Y/Beta Variant, and 346K|484K|501Y), which began emerging in November, 2020, with the exception of Beta, which according to our analysis, first appeared in July. By March, 2021, the WT had become less prevalent than the Alpha, Gamma, and Delta variants. We pursued further analysis for the Gamma and Delta variant RBDs given their high and rising global prevalence. The complexes were prepared starting from the WT crystal structures and treated identically to the WT. However, we also demonstrate that comparable results can be obtained starting directly with the native conformations approximated for the WT, which significantly reduces computational burden (see Discussion and Extended Methods in the SI Appendix).

The rapid emergence and subsequent displacement of RBD single mutants might in part result from epistasis among the RBD variant residues or from purely additive interactions. The most prominent trend was the displacement of the single mutant, 501Y (Alpha variant) by the Beta and Gamma variants (both also containing 501Y). Residue 501Y has been shown to substantially increase the binding affinity with ACE2 which, however, is reduced with the addition of mutation 417N in the Beta variant(11). In contrast, 417N severely reduces the neutralizing activity of a variety of NAb(24). These observations imply that mutations in site 417 provide a selective advantage through destabilization of the NAb complex, but given the large overlap between the RBD-NAb and RBD-ACE2 interfaces, maintenance of sufficient infectivity requires a compensatory mutation, such as 501Y, that stabilizes the RBD-ACE2 complex.

Examination of the interface footprints, defined as the ensemble of sites predicted to lie at the interface of at least one of the 50 conformations for each complex, for the 6 complexes of interest demonstrates that RBD makes a greater number of contacts with NAb than with ACE2 within the same range of sites, 403–506 (Figure 2B). The footprints of the WT and Delta variant interfaces in both the RBD-ACE2 and the RBD-NAb complexes are identical. The WT/Delta RBD-ACE2 footprint consists of 37 sites whereas the WT/Delta RBD-NAb footprint consists of 51 sites (Table 1). The Gamma RBD-ACE2 footprint consists of 41 sites including all those in the WT/Delta footprint, with the single notable exception of site 484, and five additional sites. The Gamma RBD-NAb footprint consists of 53 sites including all those in the WT/Delta interface and, in addition, sites 408 and 480. Sites in the RBD-NAb footprint that are not shared by the RBD-ACE2 footprint might provide routes for the emergence of vaccine escape variants. However, because the RBD-ACE2 footprint is smaller than the RBD-NAb interface, the former is more sensitive to perturbation than the latter, for example, from mutations in site 417, which is part of the footprint of all 6 complexes. The Gamma variant has a larger footprint with ACE2 compared to the other variants, potentially reducing this sensitivity. Notably, however, site 484 is absent from the Gamma RBD-ACE2 footprint (although Gamma variant contains mutation 484K), but remains in the RBD-NAb footprint.

When a second mutation, *M*_*j*_, is introduced in addition to a prior mutation (Figure 2C), *M*_*i*_, the resulting conformational change can be additive so that the effect of the two mutations is the sum of the effects of the two individual mutations. In this case, the position of the double-mutant *M*_*i,j*_ on the plane defined by the receptor cost and antibody cost relative to the single mutant, *M*_*i*_, will be the same as that of the single-mutant, *M*_*j*_, relative to the WT. If the conformational change is non-additive, representing an epistatic relationship, the resulting trends can be classified by their impact on potential vaccine escape. Such trends could be escape-neutral when the ensemble of candidate vaccine escape mutations differs from that for the WT, but the number of such candidates is the same; escape-minimizing when the antibody cost is on average reduced relative to the receptor cost across all mutations for the mutant vs the WT; or escape-exacerbating where the antibody cost is on average increased.

**Figure 3.**
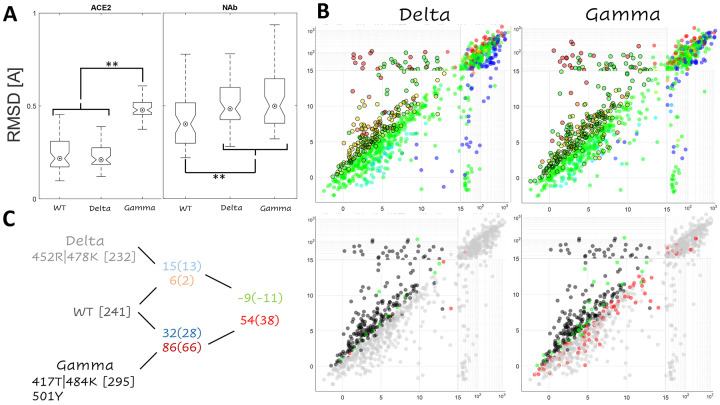
Epistasis within the RBD. **A.** Interface RMSD for NAb and ACE2 complexes relative to an arbitrary WT conformation. Asterisks denote p-values less than 0.02 for a Wilcoxon Rank Sum Test. **B.** *Top:* Landscape of vaccine escape mutations for the variant RBDs. Coloring, as in Figure 1C, indicates propensity for escape as measured by *ΔΔG*. Circles with a black outline denote NAb escape candidates. *Bottom:* Landscape of vaccine escape mutations for the WT RBD. Black points are candidates for both WT and variant; gray points are not candidates for either WT or variant; green points are only candidates for WT; red points are only candidates for the variant. **C.** Tabulation of the total number of escape candidates for the WT (241), Delta (232), and Gamma variants (295); candidates present in WT but not variant; and size difference between ensembles, Delta (−9) and Gamma (54). See main text for details.

Consistent with the differences in the footprints, we found the Gamma variant RBD conformation in complex with ACE2 to be moderately different from that of the WT and Delta variants, which could not be differentiated from one another. While both the Gamma and Delta variant RBD conformations in complex with the NAb were found to be significantly different from that of the WT, the magnitude of this difference was modest and smaller in magnitude than the variability among the RBD-NAb conformations (Figure 3A). Despite these differences, the effects of mutations in the RBD were found to be principally additive, that is, there seems to be little epistasis (Figure 3B, see Extended Methods in the SI Appendix).

The landscape of mutants predicted to enhance vaccine escape for the Delta variant was almost identical to that of the WT but differed significantly from the Gamma variant landscape. Mutations in the Gamma variant tend to have a lower receptor cost relative to the WT, possibly, due to the larger RBD-ACE2 footprint (see above), resulting in an increased number of escape candidates. These trends are summarized in Figure 3C, which tabulates all non-shared candidates. There are 15(13) escape candidates in the WT that were not predicted to enhance escape for Delta and 6(2) candidates in Delta but not WT (values in parentheses are mutations with ${\left[S_{M}^{C}-S_{WT}^{C}\right]}_{ACE2}<13$, regardless of whether or not the mutation is a candidate, included to mitigate potential artifacts caused by steric or charge clashes). In contrast, in the case of Gamma, there were 32(28) candidates identified in WT but not Gamma, and 86(66) candidates identified in Gamma but not the WT. Thus, we identified 9(11) fewer candidates for Delta compared to the WT but 54(38) additional candidates for Gamma.

**Figure 4.**
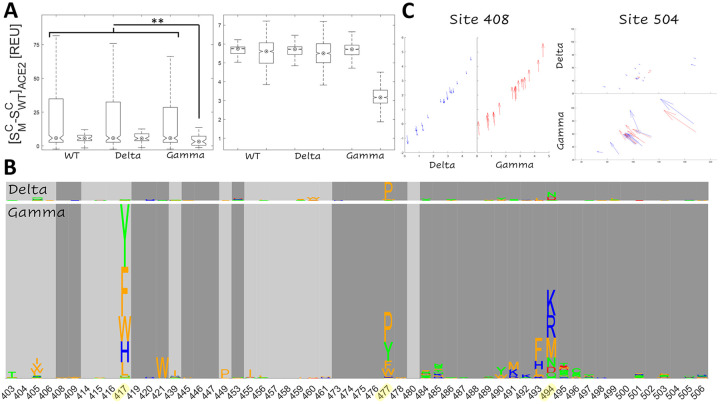
Landscape of Non-Additive Mutation Effects Supporting Enhanced Vaccine Escape for the Gamma Variant **A.***Left:* Receptor cost for mutations at the interface that were experimentally demonstrated to reduce NAb activity in the WT(8) (right) and other experimentally studied mutations at the interface (left). Asterisks represent p-values less than 0.02 for a Wilcoxon Rank Sum Test. *Right:* Distribution of median values over 1000x bootstrap. **B.** Non-additive escape-exacerbating motifs in Delta (top) and Gamma (bottom) variants. The size of each letter corresponds to the increased likelihood of vaccine escape for the substitution in the variant relative to the WT. **C.** Movement within the plane of receptor cost vs. antibody cost. Each arrow represents an amino acid substitution in site 408 or 504, and arrows point from the position on the plane corresponding to substitution in the WT to the position corresponding to the substitution in each variant. Color represents the sign of *ΔΔG*_*NAb*_ – *ΔΔG*_*ACE2*_ (blue, negative; red, positive).

Consistent with the more dramatic conformational change observed in the RBD-ACE2 complex relative to the RBD-NAb complex, the non-additive effects observed in the Gamma variant appear to predominantly result from the decreased sensitivity of the RBD-ACE2 interface to mutation. This conclusion is compatible with the available experimental results. Figure 4A shows the distribution of the receptor cost, ${\left[S_{M}^{C}-S_{WT}^{C}\right]}_{ACE2}$, for two categories of mutations for each of the three receptor complexes: those at the interface that have been experimentally demonstrated to reduce neutralizing activity of antibodies COV2–2050 and COV2–2479 in the WT(8), and all others (included in the same experimental study) at the interface. As discussed above, the upper bound for the receptor cost, ${\left[S_{M}^{C}-S_{WT}^{C}\right]}_{ACE2}$, is lower for mutations predicted to reduce NAb activity than for other mutations. However, only the Gamma candidate ensemble exhibits a reduced median receptor cost (see bootstrap analysis in Figure 4A). In other words, in the Gamma variant, mutations that are predicted to reduce NAb activity are also less likely than other mutations to reduce the receptor binding affinity relative to the WT and Delta variant.

Figure 4B summarizes the magnitude of the increase of the risk of vaccine-escape for each mutation at the RBD interface (given ${\left[S_{M}^{C}-S_{WT}^{C}\right]}_{ACE2}<13$). Epistasis increasing the risk of vaccine-escape is apparent in three regions of the RBD interface: site 417, site 477, and site 494 together with the surrounding neighborhood. The trend in site 417 was observed only in Gamma, which already contains mutation 417T, showing that further changes to this site could result in enhanced vaccine escape. However, the epidemiological implications of this finding are limited considering that mutations in site 417 are likely to pose a risk of vaccine escape in most variants. The enhanced escape associated with mutations in site 477 for both variants relative to the WT, together with the early spread of 477N, suggest that this site could play an important role in future host adaptation. Most prominently, mutations in site 494 and the surrounding neighborhood are likely to enhance vaccine escape in Gamma (and to a much lesser degree in Delta). Indeed, 494P has both been found in circulation and experimentally demonstrated to reduce antibody neutralization capacity of convalescent sera(25).

In addition to these apparent differences among the ensembles of candidate vaccine-escape mutations, we observed sites that harbored no candidates but nevertheless displayed signatures of increased risk of vaccine escape for Gamma. The two most notable trends were observed in sites 408 and 504 (Figure 4C). Axes represent antibody cost vs receptor cost as in Figures 1C and 3B. All but one substitution in site 408 enhance vaccine-escape for Gamma, but strikingly, all have the opposite effect in Delta. Similarly, all substitutions at site 504 substantially enhance vaccine-escape in Gamma, but exert a modest opposite effect in Delta. However, these mutations are not considered candidates in our analysis because, even in the case of Gamma, they destabilize the ACE2 interaction to a greater extent than the interaction with NAb. Additionally, ${\left[S_{M}^{C}-S_{WT}^{C}\right]}_{ACE2}\gg 13$ for substitutions at site 504, which limits confidence in the assessment of trends at this site.

## Discussion

Here we report the results of a computational study predicting the effects of all single mutants at the RBD-NAb and RBD-ACE2 interfaces for the WT, Gamma variant (417T, 484K, 501Y), and Delta variant (452R, 478K) of SARS-CoV-2 on receptor and antibody binding. For the WT, we found multiple mutations in 6 sites (417, 477, 484, 491, 493, 499) that are predicted to significantly destabilize the RBD-NAb complex relative to the RBD-ACE2 complex and appear to pose a risk of vaccine escape, which is broadly consistent with the results of deep mutational scanning(8, 9, 19–22). Overall, most mutations at the interface were found to similarly effect the WT and both variants indicating limited epistasis at the interface. Non-additive, epistatic interactions predicted to increase the risk of vaccine-escape were apparent, however, at site 477 and site 494 as well as in the surrounding neighborhood. This trend is particularly prominent in the Gamma variant so that, across all sites at the interface, we predicted 22% more escape candidate mutations for the Gamma variant than for the WT. In contrast, there is little apparent epistasis in the Delta variant, and across all sites at the interface, we predicted 4% fewer candidate mutations compared to WT. This information potentially could be leveraged when making decisions as to which variants warrant tailored vaccine development. In addition to the observed differences in infectivity and/or vaccine resistance, variants, such as Gamma, that apparently have an easier access to vaccine escape than other variants seem to merit closer surveillance.

Epistasis is a major if not the principal driver of protein evolution(26). Compensatory mutations are particularly strong epistatic interactions that can result in chemotherapeutic(27) or antimicrobial(28) drug resistance and are commonly observed throughout species evolution(29). In a completely susceptible population, mutation 501Y, which likely substantially increases infectivity(11), is expected to evolve under positive selection. As a population gains immunity, through prior exposure and/or vaccination, selective pressures rapidly change to promote the emergence of resistant variants(7). Under these conditions, 501Y and other mutations, which increase infectivity, might primarily play the role of compensators for mutations destabilizing NAb interactions, such as 417T. As global vaccinations rise, it can be expected that more mutations emerge that destabilize the interactions of the RBD with both NAb and ACE2, thus resulting in (partial) escape variants that, however, also have reduced infectivity. However, variants such as Gamma that carry both an antibody destabilizing mutation and a compensatory mutation have the potential to undercut this trend.

We conducted structural modelling for the WT, Gamma, and Delta variants to approximate an ensemble of native conformations using a fairly expensive computational approach. While massively parallelizable, and in principle requiring less than 72 hours to compute, this method has practical limitations. To address this issue, we computed alternative ensembles of both variant conformations beginning with the WT ensemble (a computationally cheap approach) and demonstrated satisfactory agreement with the results obtained with the original protocol (see Extended Methods in the SI Appendix). With this modified approach, given the WT conformations reported in this work, the vaccine-escape landscape can be cheaply and rapidly established for any emergent variant of concern.

### Limitations

The work presented here is strictly computational, and although we demonstrate agreement with experimental results where possible, many features not captured by the models presented (involving protein expression, docking, and other factors) could modulate antigen-receptor or antigen-antibody binding. Furthermore, although we explore many conformations for both the RBD-ACE2 and RBD-NAb interfaces, we start from a single crystal structure for each. We believe the conformational ensembles selected to represent each complex are diverse enough to accurately reflect the relative destabilization of the NAb and ACE2 complexes across the spectrum of RBD interface mutations, which is of primary concern. However, this conformational diversity makes it difficult to demonstrate stabilizing interactions, which are typically much weaker than destabilizing ones(12). While multiple low-energy conformations were resolved for each variant and the WT, the average behavior of the conformational ensemble selected to represent the Delta variant relative to the WT was found to be weakly destabilizing for NAb and neutral for ACE2, whereas in the case of Gamma, it was weakly destabilizing for both complexes. This is unlikely to accurately reflect the relative binding affinities between these variants and the WT given the enhanced infectivity of both variants, particularly Delta(14). However, it is important to recognize that the relationship between the measures of interface stability we report and viral life history traits (infectivity, immune activity, etc.) is complex. Although we believe we proposed sensible thresholds for determining which structures can be analyzed with high confidence and the biological implications of the relative destabilization of the NAb vs ACE2, the effects studied in this work do not represent the diversity of possible host adaptation. It is incompletely understood at the time of writing why the Delta variant appears to replicate faster than the WT(14) and substitutions outside the Spike protein may play key roles in immune modulation(30, 31). A targeted exploration of the lowest-energy conformations achievable for each variant might yield better agreement with the known properties of these variants. However, this would likely come at the cost of generalizability and decrease the power with which our approach is able to predict relative destabilization of interface mutations between NAb and ACE2 complexes.

### Conclusions

We employed a computational approach to study the effects of all single mutations at the RBD-NAb and RBD-ACE2 interfaces for the WT, Gamma variant (417T, 484K, 501Y), and Delta variant (452R, 478K) of SARS-CoV-2. Overall, little epistasis at the RBD interface was detected, with additive effects on the binding affinities observed for most pairs of mutations. In the Delta variant, the detected non-additive trends weakly stabilize the interaction of the RBD with the NAb relative to the interaction with ACE2, whereas in the Gamma variant, epistasis is predicted to more substantially destabilize interaction with the NAb relative to ACE2. These results suggest that the mutational repertoire of the Delta variant is slightly less prone to the emergence of new escape mutations than that of the WT, whereas the Gamma variant poses a moderately greater risk of enhanced vaccine escape. The modest ensemble of mutations relative to the WT that are currently known to reduce vaccine efficacy is likely to comprise the majority of all possible escape mutations for future variants.

## Brief Methods

The RBD-ACE2 interface was modelled starting with the crystal structure of the RBD in complex with ACE2 https://www.rcsb.org/structure/6M0J(16). Residues outside the RBD that might affect binding (e.g. 614(32)) were not considered. The RBD-NAb interface was modelled starting with the crystal structure of the respective complex: https://www.rcsb.org/structure/6XE1(33, 34). Epitopes outside the RBD that might be epidemiologically relevant (35, 36) were not considered. The Rosetta(13, 37) software suite was used to approximate native conformations(38) relative to the crystal structure through a process of backbone minimization and side chain repacking. The total score, *S*, in arbitrary units(18) (labelled REU for “Rosetta Energy Units”, https://new.rosettacommons.org/docs/latest/rosetta_basics/Units-in-Rosetta), as well as *ΔG* separated, which is the difference in the total score between the bound (complex) and unbound state, *S*^*C*^−*S*^*U*^, derived from separating the binding partners were used to assess interface stability. A lower total score, *S*^*C*^, and lower *ΔG* separated, *S*^*C*^−*S*^*U*^*<0* indicate relative stability. We first searched for an “energy funnel”(39) among the approximated native conformations. However, a funnel was not identified, so we proceeded to select an ensemble of conformations (50 per interface) based on a total score and *ΔG* separated ranking protocol to represent each interface rather than the single lowest-energy conformation, which may be an entropically disfavored state(40). We then predicted 52 residues to lie at the interface of at least one structure for WT/Delta and 56 for Gamma. We introduced each of the19 possible mutations at each interface site for these 100 structures for the WT, Delta, and Gamma variants, with the exception of the WT reversion for the variants. Mutations were introduced without repacking and minimization for a maximally computationally efficient approach. Alternative approaches have been reviewed (41–44), but the minimal approach pursued maximizes the breadth of candidate mutations considered and is able to recapitulate experimental results. As an alternative to constructing the variant ensembles starting with the crystal structure, we considered starting with the conformational ensemble constructed for the WT and applying a reduced protocol of iterative minimization and repacking for conformational optimization. This approach produced results compatible with those obtained with the original protocol and can be utilized for rapid evaluation of emerging variants of concern (see Extended Methods in the SI Appendix).

## Supplementary Material

Supplement 1

## Figures and Tables

**Table 1:** Receptor-binding and antibody-binding interface footprints in the RBD

WT RBD-ACE2 Footprint	403, 405, 417, 445–447, 449, 453, 455–456, 473–478, 484–491, 493498, 500–506
Delta RBD-ACE2 Footprint	Same as WT
Gamma RBD-ACE2 Footprint	WT + 404, 406, 408, 439, 499 – 484
WT RBD-NAb Footprint	403–406, 409, 414–417, 419–421, 446–447, 449, 453, 455–461, 473478, 484–498, 500–506
Delta RBD-NAb Footprint	Same as WT
Gamma RBD-NAb Footprint	WT + 408, 480

## Data Availability

The data has been deposited through Zenodo(45) (https://doi.org/10.5281/zenodo.5297699) including GISAID acknowledgements. Previously published data were used for this work: GISAID(23). Data is additionally made available through FTP: https://ftp.ncbi.nih.gov/pub/wolf/_suppl/SARSstruct21/

## Significance

Potential emergence of vaccine escape variants of SARS-CoV-2 is arguably the most pressing problem during the COVID-19 pandemic as vaccines are distributed worldwide. We employed a computational approach to assess the risk of antibody escape resulting from mutations in the receptor-binding domain of the spike protein of the wild type SARS-CoV-2 virus as well as the Gamma and Delta variants. The results indicate that emergence of escape mutants is somewhat less likely for the Delta variant than for the wild type and moderately more likely for the Gamma variant. We conclude that the small set of escape-enhancing mutations already identified for the wild type is likely to include the majority of all possible mutations with this effect, a welcome finding.
