# Supplementary material for: Epistasis at the SARS-CoV-2 RBD Interface and the Propitiously Boring Implications for Vaccine Escape
Source: bioRxiv. 2021 Aug 30:2021.08.30.458225. Preprint. [Version 1] doi: 10.1101/2021.08.30.458225 (PMC8423221; doi:10.1101/2021.08.30.458225)
Supplement: Supplementary file 1 [file media-1.pdf]

PNAS
www.pnas.org

Supplementary Information for

Epistasis at the SARS-CoV-2 RBD Interface and the Propitiously Boring Implications for Vaccine Escape

Nash D. Rochman, Guilhem Faure, Yuri I. Wolf, Peter, Peter L. Freddolino, Feng Zhang, and Eugene V. Koonin

Nash D. Rochman
Email: nash.rochman@nih.gov

Feng Zhang
Email: zhang@broadinstitute.org

Eugene V. Koonin
Email: koonin@ncbi.nlm.nih.gov

This PDF file includes:

Extended Methods
Figures S1 to S12
Table S1

Additional supplementary information can be found:

https://ftp.ncbi.nih.gov/pub/wolf/_suppl/SARSevo21/
or
https://doi.org/10.5281/zenodo.5297699

This repository includes:

GISAID Acknowledgements
An example resfile
RosettaScripts .xml files
Complex conformations (50 each) for WT/Gamma/Delta; antibody (NAb) and receptor (ACE2)

Extended Methods

Selection of Crystal Structures

In this work, we considered a single crystal structure of the SARS-CoV-2 spike protein Receptor Binding Domain (RBD) in complex with the human receptor, Angiotensin Converting Enzyme 2 (ACE2), PDB:6M0J(1). While there are likely multiple mutations outside of the RBD which significantly affect binding characteristics of the spike protein, as has been demonstrated for site 614(2), the structure of the RBD itself is unlikely to be substantially modified by such mutations. Thus, while only able to reveal a subset of mutations of interest, the focused study of RBD complexes presented here remains biologically realistic.

Similarly, we consider a single crystal structure of the RBD in complex with a neutralizing antibody (NAb),CV30 PDB:6XE1(3). CV30 was recognized early on as the most potent NAb observed within the sera of a SARS-CoV-2 positive donor while the majority of antibodies were found to target non-neutralizing epitopes outside of the RBD(4). Subsequently, it became clear that antibodies targeting epitopes outside the RBD, particularly those targeting the N-terminal domain (NTD), likely play a protective role and mutations reducing the affinity of these antibodies may be epidemiologically significant(5, 6). As acknowledged above regarding the selection of the spike crystal structure, while our focus on CV30 is only able to reveal a subset of mutations of interest, the mutations discussed in this work predicted to affect CV30 binding affinity are likely highly epidemiologically relevant.

Construction of Representative Ensembles of Interface Conformations

Protein crystal structures may differ substantially from the native conformations(7). Throughout this work, we utilize the Rosetta(8) software suite to approximate both wild type (WT) and mutant conformations of the receptor and NAb complexes. All protocols used throughout were implemented using the RosettaScripts(9) package and the XML files used along with the associated executed command lines are made available in Table S1. Approximation of the native conformational ensemble may be separated into two steps, identifying the optimal side conformation (repacking) and moving the protein backbone (minimization), to minimize the energy function applied. This may be accomplished using the Rosetta Relax application which iteratively applies each of these two steps.

Beginning with the crystal structure, we iteratively applied the FastRelaxMover using default parameters (with the exception of disabling design) for up 12 iterations and up to 1000 repeats. We found the total score to be insensitive to additional applications of FastRelax after 5 iterations on average. Each resulting structure was scored using the InterfaceAnalyzerMover, repacking the unbound state but not the bound state (as the input complex has already been optimized).

This protocol returns the total score, *S*, in arbitrary units produced by the empirically-driven Rosetta Energy Function 2015(10) (labelled REU for “Rosetta Energy Units”, https://new.rosettacommons.org/docs/latest/rosetta_basics/Units-in-Rosetta), as well as dG separated which is the difference in the total score between the bound (complex) and unbound state, S^C-S^U, derived from separating the binding partners. This protocol may also be used to identify the residues within the complex which constitute the interface.

Such an ensemble of structures often forms an “energy funnel”(11) where the root mean square distance (RMSD) between superimposed backbone carbon atoms of each structure and the structure with the lowest total score or the lowest dG separated is positively correlated with the total score of that structure. In order to evaluate whether such a funnel exists for these ensembles, we identified the structure with the lowest dG separated and 90 residues predicted to be interface residues within the RBD in at least one conformation in addition to the (+/-) 3 adjacent amino acid neighborhood of each such residue (sites 400-424, 440-464, and 470-509 in the spike protein for WT; additional sites 436-439 for Delta; additional sites 434-439 for Gamma).

Figure S2 displays dG separated vs the interface RMSD for both complexes for the WT. Few structures appear in the lower left corner of each plot from only one or two (NAb or ACE2 respectively) independent “trajectories” of iterative FastRelax application beginning with the crystal structure. Furthermore, while the lowest dG separated is more than 30% greater in magnitude than the highest for both complexes, the interface RMSD between any complex and the minimum dG separated complex is less than an angstrom. These findings suggest selecting the single minimum dG separated conformation for either complex is unlikely to constitute a realistic model of the native interface and may in fact represent an unrealistic, entropically disfavored state(12).

Figures S3-4 display dG separated vs the interface RMSD for both complexes for the Delta and Gamma variants. The distribution of conformations for the NAb is similar to the WT for both Delta and Gamma; however, the minimum dG separated obtained for Gamma is higher than that for the WT suggestive of antibody destabilization for this variant. For Delta, the distribution of conformations for ACE2 more resembles a funnel suggestive of receptor stabilization for this variant. For Gamma, there appear to be 2-3 distinct, equally low energy conformations for ACE2 which makes the interpretation of the energy landscape more challenging. For consistency, representative conformations were selected according to the same protocol for the WT, Delta, and Gamma variants.

The construction of an ensemble of representative conformations is desired so that the average behavior of such an ensemble is likely to reflect that of the native complex. Ideally all available structures would be statistically weighted and included in this ensemble; however, this is computationally intractable. Instead we selected 50 conformations for each complex as follows (see Figs. S5-7): 1) The conformation corresponding to the minimum total score after any iteration of FastRelax from each independent “trajectory” beginning with the crystal structure was selected. 2) The 10

structures with the lowest total score were removed and the 10 structures with the lowest dG separated were removed (these are not identical structures and as discussed above, may represent unrealistic, entropically disfavored conformations). 3) The remaining structures were ranked by total score, r_S, and dG separated, r_G, and the 50 structures with the lowest composite rank, r_S+r_G, were selected. The PDB files for these structures are made available (see Data Availability). We believe the equally-weighted average behavior of these conformations, which are stable with well-resolved interfaces, constitute a reasonable model of the native complexes.

Analysis of Single Mutants Relative to WT, Delta, and Gamma

Given the 100 structures selected as described above (50 for each complex), a more restrictive list of 52 residues were predicted to lie at the interface of at least one structure for WT/Delta (spike protein sites 403-406,409,414-417,419-421,445-447, 449,453,455-461,473-478,484-498,500-506) and 56 for Gamma (WT/Delta+408,439,480,499).

We introduced all of the 19 possible mutations at each interface site for these 100 structures for the WT, Delta, and Gamma variants with the exception of WT reversion for the variants. Mutations were introduced using the PackRotamersMover with design specified by the input resfile (see Data Availability for example). Only the targeted residue was modified: side chain conformations for all other residues were fixed and no backbone minimization was applied before filtering for candidates of interest as described below.

Introducing the mutation in this way without repacking and minimization results in many unrealistic, high-energy conformations which are difficult or impossible to interpret without further optimization. The benefit is speed. This procedure can be executed in under 30 seconds per structure while global repacking and minimization may take hours. An alternative approach is to apply backbone minimization and sidechain repacking within a local region of the structure centered at the modified site as implemented within Rosetta through the Flex ddG protocol(13). This approach has been used to accurately predict mutations conferring increased infectivity for SARS-CoV-2(14) and rationally design NAb against the antigen(15, 16) but is significantly slower than introducing mutations without, even local, optimization. With or without local optimization prior to filtering, global repacking and minimization is desired to confirm top candidates. While our approach may introduce a higher false-negative rate than what one would achieve applying local optimization prior to filtering, it maximizes the breadth of candidate mutations considered and is able to recapitulate experimental results.

The total score and dG separated of the mutant structures were computed. Unlike in earlier steps where the unbound state was repacked during the dG separated calculation, no repacking was conducted for the reasons described above. Each mutant structure was matched with its initial WT/variant conformation and the change in dG

separated, $(S_M^C\text{-}S_M^U)\text{-}(S_{WT}^C\text{-}S_{WT}^U)$, and total score, $S_M^C\text{-}S_{WT}^C$, were calculated. The average of each value taken over the ensemble of the 50 RBD-ACE2 conformations was compared with experimentally determined ACE2 binding affinity for single RBD mutants(17). While the change in dG separated is technically the ddG for the mutant and may be expected to correspond to binding affinity, we find $(S_M^C\text{-}S_M^U)\text{-}(S_{WT}^C\text{-}S_{WT}^U)$ over this ensemble is not generally predictive of ACE2 affinity as $(S_M^C\text{-}S_M^U)\text{-}(S_{WT}^C\text{-}S_{WT}^U)$ is approximately zero for many mutants (Fig. S8). This may be expected without repacking and minimization. Nonetheless, when nonzero, the change in dG separated compares favorably with ACE2 affinity (see below).

The change in total score is less sensitive and an adequate predictor of affinity (Fig. S9) within the range $S_M^C\text{-}S_{WT}^C<5$ such that the minimum relative binding affinity decreases with increasing change in total score. Additional validation that $S_M^C\text{-}S_{WT}^C$ is a reasonable predictor of binding affinity, below some threshold, may be obtained through demonstrating $S_M^C\text{-}S_{WT}^C$ for each mutant is highly correlated between the RBD-ACE2 complex and the RBD-NAb complex. Furthermore, when the difference between the change in dG separated for the NAb complex and the dG separated for the ACE2 complex, $[(S_M^C\text{-}S_M^U)\text{-}(S_{WT}^C\text{-}S_{WT}^U)]_{NAb}\text{-}[(S_M^C\text{-}S_M^U)\text{-}(S_{WT}^C\text{-}S_{WT}^U)]_{ACE2}$, is positive, mutants lie above the identity line (y==x). The reverse is true for mutants falling below the identity line (Figures 1C/3B). In other words, on average, mutants in the RBD-ACE2 complex behave similarly to mutants in the RBD-NAb complex (which is what one would expect for realistic models of interfaces with an overlapping footprint) and when differences do appear, they correspond to interactions with the RBD which are specific to the binding partner (ACE2/NAb); and are correctly reflected by the change in dG separated.

Directly Assessing Additive Effects Vs Epistasis

In the main text, we demonstrate that most mutations appear in a similar position of the receptor cost / antibody cost phase space for the WT and Gamma/Delta variants. Consequently, the ensemble of vaccine escape candidates is largely conserved. Whether epistatic effects due to multiple mutations are present or if the impact of multiple mutations is simply additive can be more directly assessed for each complex by plotting the change in the total score after introducing each mutation in the variant against the change in total score after introducing each mutation in the WT. Figure S10 displays the change in total score for each interface mutation relative to the WT and each variant for both ACE2 and the NAb. The (minimum value – 1) is subtracted from each distribution. The effect of each mutation at the interface in the WT is highly correlated with each variant with greater variability for Gamma than Delta as expected.

Alternative Construction of Variant Ensembles

Beginning with the conformational ensemble constructed for the WT, we introduced the variant mutations 452R|478K for the Delta variant and 417T|484K|501Y for the Gamma

variant and completed 5 rounds of the FastRelaxMover using default parameters (with the exception of disabling design) as described above. The resulting values for the total score and dG separated were comparable to that of the ensembles constructed for the variants beginning with the WT crystal structure (Figures S11-12). Thus this computationally cheap alternative may be utilized for rapid evaluation of emerging variants of concern.

Supplemental Figures

Figure S1. Number of escape candidates and the mean score for each candidate, *max(*$[S_M^C - S_{WT}^C]_{NAb} - [S_M^C - S_{WT}^C]_{ACE2}$, ddG_{NAb}-ddG_{ACE2}*)*, across all RBD Interface sites for the WT.

Figure S2. dG separated vs. the interface RMSD with the structure corresponding to the lowest dG separated for the WT computed over the alpha carbon backbone atoms of identified interface residues and the associated (+/-) 3 amino acid neighborhoods of each in the RBD (spike protein sites 400-424, 440-464, and 470-509).

Figure S3. dG separated vs. the interface RMSD with the structure corresponding to the lowest dG separated for the Delta variant computed over the alpha carbon backbone atoms of identified interface residues and the associated (+/-) 3 amino acid neighborhoods of each in the RBD (spike protein sites 400-424, 436-464, and 470-509).

Figure S4. dG separated vs. the interface RMSD with the structure corresponding to the lowest dG separated for the Gamma variant computed over the alpha carbon backbone atoms of identified interface residues and the associated (+/-) 3 amino acid neighborhoods of each in the RBD (spike protein sites 400-424, 434-464, and 470-509).

Figure S5. dG separated vs. the total score for WT conformations achieved after iterative applications of FastRelax beginning with the crystal structure. Blue points represent iterations 1-4 and green represent iterations 5-12. Points outlined in black are the 50 conformations selected to represent each complex.

Figure S6. dG separated vs. the total score for Delta variant conformations achieved after iterative applications of FastRelax beginning with the crystal structure. Blue points represent iterations 1-4 and green represent iterations 5-12. Points outlined in black are the 50 conformations selected to represent each complex. Gray dots are WT conformations.

Figure S7. dG separated vs. the total score for Gamma variant conformations achieved after iterative applications of FastRelax beginning with the crystal structure. Blue points represent iterations 1-4 and green represent iterations 5-12. Points outlined in black are the 50 conformations selected to represent each complex. Gray dots are WT conformations.

Figure S8. Relative ACE2 affinity as reported by Starr et. al(17), positive values indicate stronger affinity, vs difference in dG separated between the mutant and the WT.

Figure S9. Relative ACE2 affinity as reported by Starr et. al(17), positive values indicate stronger affinity, vs difference in total score between the mutant and the WT.

Figure S10. Total score change induced by mutation in each variant vs the WT. The (minimum value – 1) is subtracted from each distribution.

Figure S11. dG separated vs total score for the original Delta variant conformational ensemble generated and the alternate ensemble.

Figure S12. dG separated vs total score for the original Gamma variant conformational ensemble generated and the alternate ensemble.

Supplemental Tables

Table S1. RosettaScripts files, descriptions, and example command line implementations.

References

1. Lan J, *et al.* (2020) Structure of the SARS-CoV-2 spike receptor-binding domain bound to the ACE2 receptor. *Nature* 581(7807):215-220.
2. Zhang J, *et al.* (2021) Structural impact on SARS-CoV-2 spike protein by D614G substitution. *Science* 372(6541):525-530.
3. Hurlburt NK, *et al.* (2020) Structural basis for potent neutralization of SARS-CoV-2 and role of antibody affinity maturation. *Nature communications* 11(1):1-7.
4. Seydoux E, *et al.* (2020) Analysis of a SARS-CoV-2-infected individual reveals development of potent neutralizing antibodies with limited somatic mutation. *Immunity* 53(1):98-105. e105.
5. Voss WN, *et al.* (2021) Prevalent, protective, and convergent IgG recognition of SARS-CoV-2 non-RBD spike epitopes. *Science* 372(6546):1108-1112.
6. Garushyants SK, Rogozin IB, & Koonin EV (2021) Insertions in SARS-CoV-2 genome caused by template switch and duplications give rise to new variants of potential concern. *bioRxiv*.
7. Tyka MD, *et al.* (2011) Alternate states of proteins revealed by detailed energy landscape mapping. *Journal of molecular biology* 405(2):607-618.
8. Leaver-Fay A, *et al.* (2011) ROSETTA3: an object-oriented software suite for the simulation and design of macromolecules. *Methods in enzymology* 487:545-574.
9. Fleishman SJ, *et al.* (2011) RosettaScripts: a scripting language interface to the Rosetta macromolecular modeling suite. *PloS one* 6(6):e20161.
10. Alford RF, *et al.* (2017) The Rosetta all-atom energy function for macromolecular modeling and design. *Journal of chemical theory and computation* 13(6):3031-3048.
11. Tovchigrechko A & Vakser IA (2001) How common is the funnel-like energy landscape in protein-protein interactions? *Protein science* 10(8):1572-1583.

12. Grünberg R, Nilges M, & Leckner J (2006) Flexibility and conformational entropy in protein-protein binding. *Structure* 14(4):683-693.
13. Barlow KA*, et al.* (2018) Flex ddG: Rosetta ensemble-based estimation of changes in protein–protein binding affinity upon mutation. *The Journal of Physical Chemistry B* 122(21):5389-5399.
14. Xue T*, et al.* (2021) Single point mutations can potentially enhance infectivity of SARS-CoV-2 revealed by in silico affinity maturation and SPR assay. *RSC Advances* 11(24):14737-14745.
15. Riahi S*, et al.* (2021) Application of an integrated computational antibody engineering platform to design SARS-CoV-2 neutralizers. *bioRxiv*.
16. Desautels T, Zemla A, Lau E, Franco M, & Faissol D (2020) Rapid in silico design of antibodies targeting SARS-CoV-2 using machine learning and supercomputing. *BioRxiv*.
17. Starr TN*, et al.* (2020) Deep mutational scanning of SARS-CoV-2 receptor binding domain reveals constraints on folding and ACE2 binding. *Cell* 182(5):1295-1310. e1220.

S1

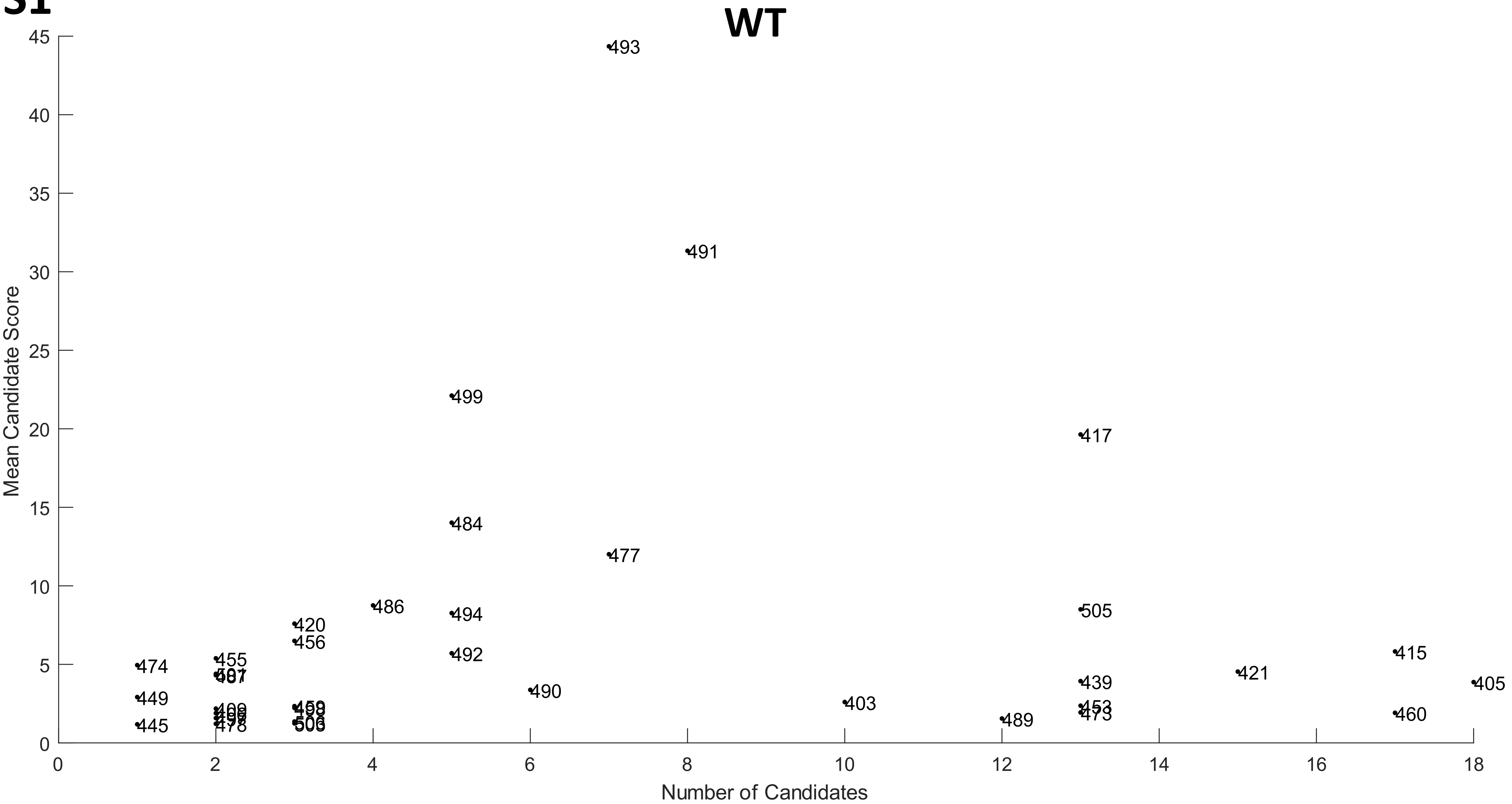
WT
Mean Candidate Score
Number of Candidates
0
5
10
15
20
25
30
35
40
45
0
2
4
6
8
10
12
14
16
18
493
491
499
417
484
477
486
505
420
494
456
492
474
455
415
421
439
405
490
449
403
460
489
445
473
478

S2

WT

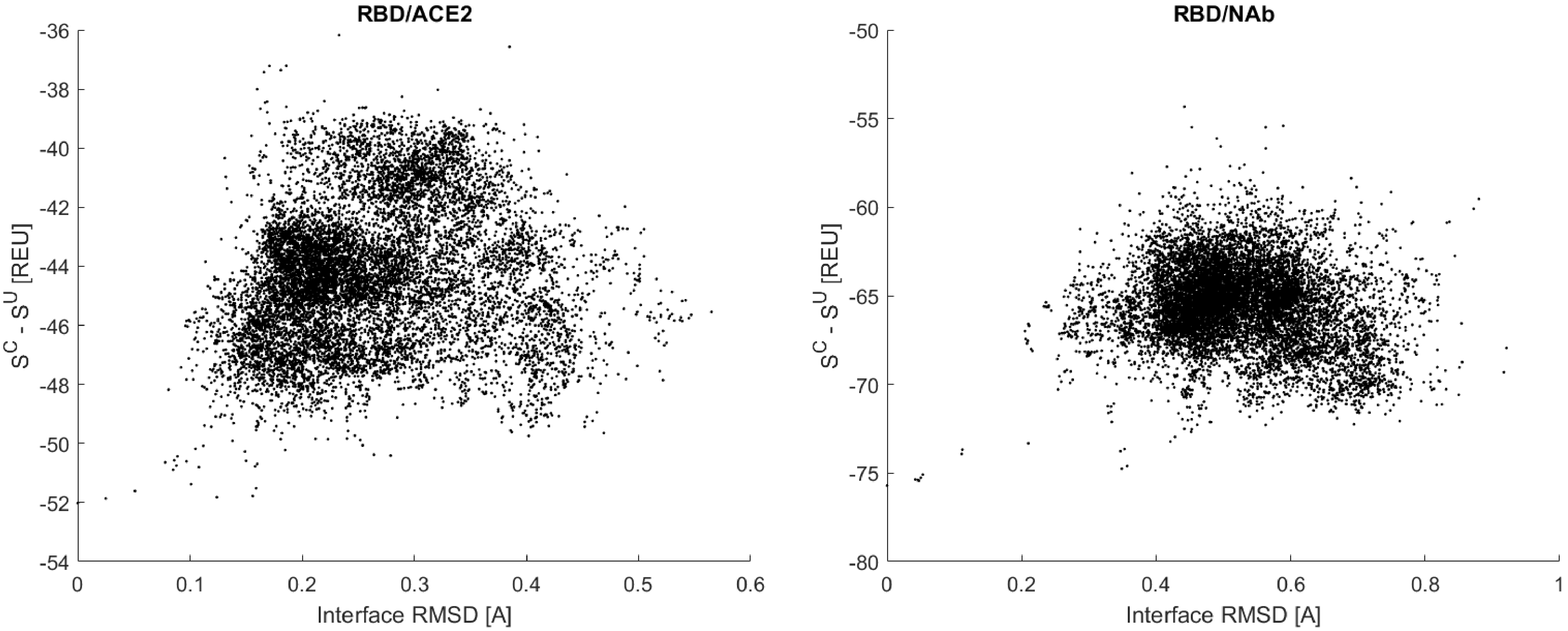
RBD/ACE2
-36
-38
-40
-42
-44
-46
-48
-50
-52
-54
$S^C - S^U$ [REU]
0
0.1
0.2
0.3
0.4
0.5
0.6
Interface RMSD [A]
RBD/NAb
-50
-55
-60
-65
-70
-75
-80
$S^C - S^U$ [REU]
0
0.2
0.4
0.6
0.8
1
Interface RMSD [A]

S3

Delta

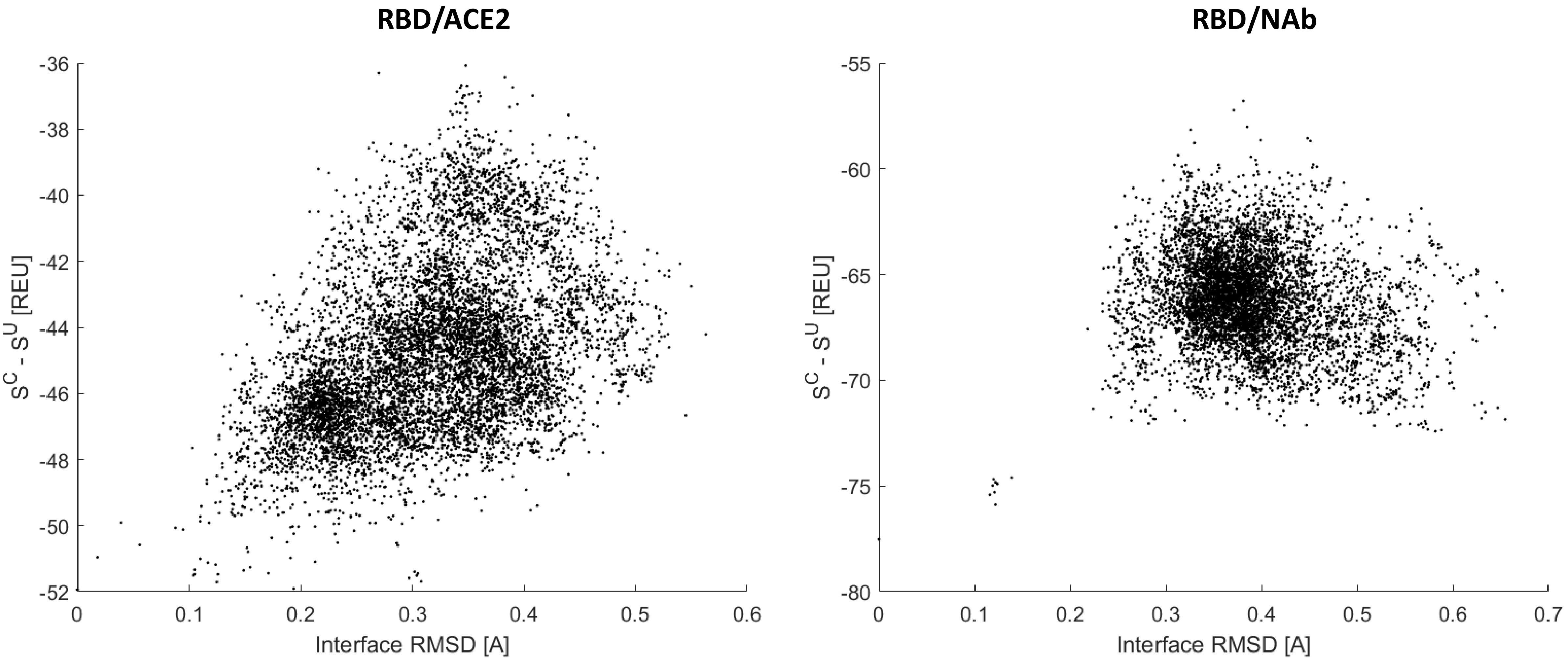
RBD/ACE2
-36
-38
-40
-42
-44
-46
-48
-50
-52
S^C - S^U [REU]
0
0.1
0.2
0.3
0.4
0.5
0.6
Interface RMSD [A]
RBD/NAb
-55
-60
-65
-70
-75
-80
S^C - S^U [REU]
0
0.1
0.2
0.3
0.4
0.5
0.6
0.7
Interface RMSD [A]

S4

Gamma

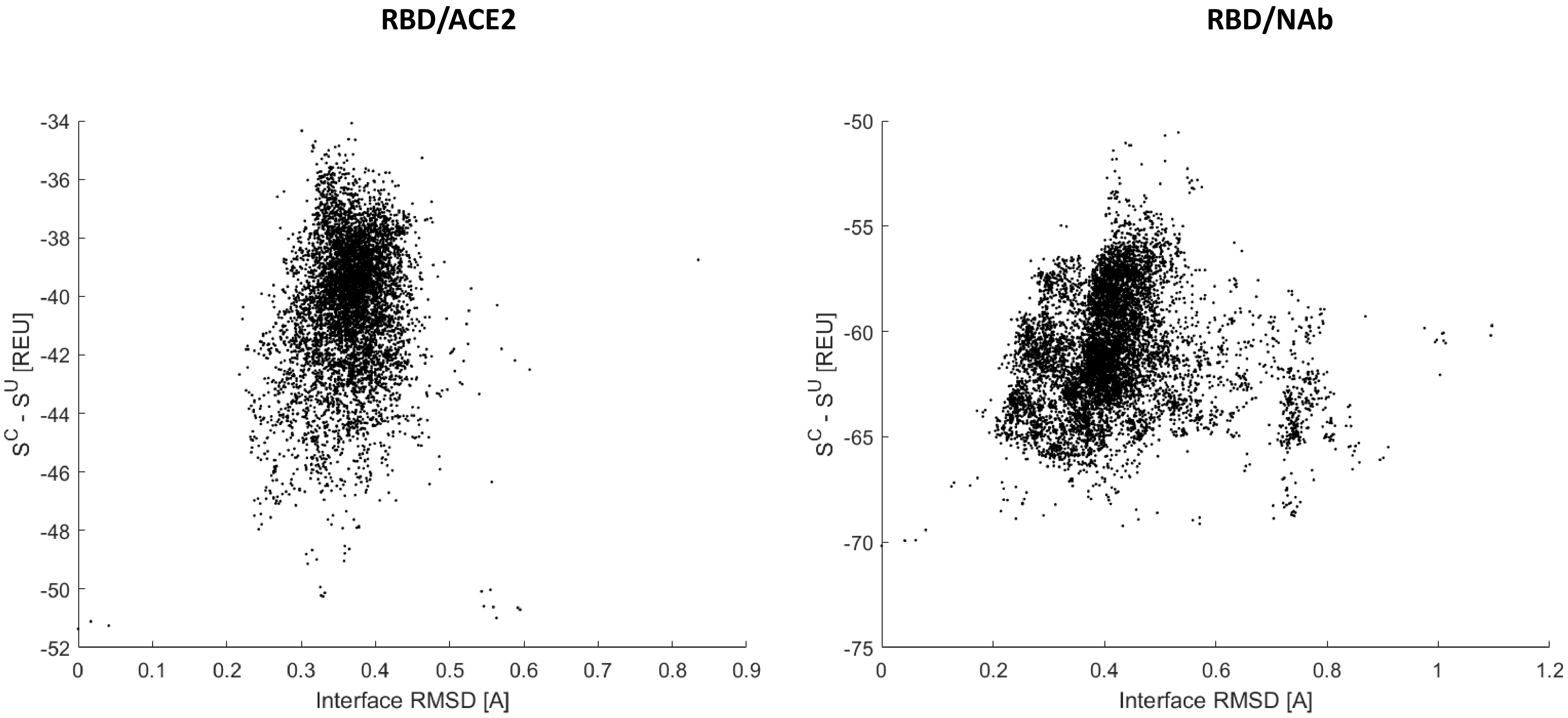

S5

WT

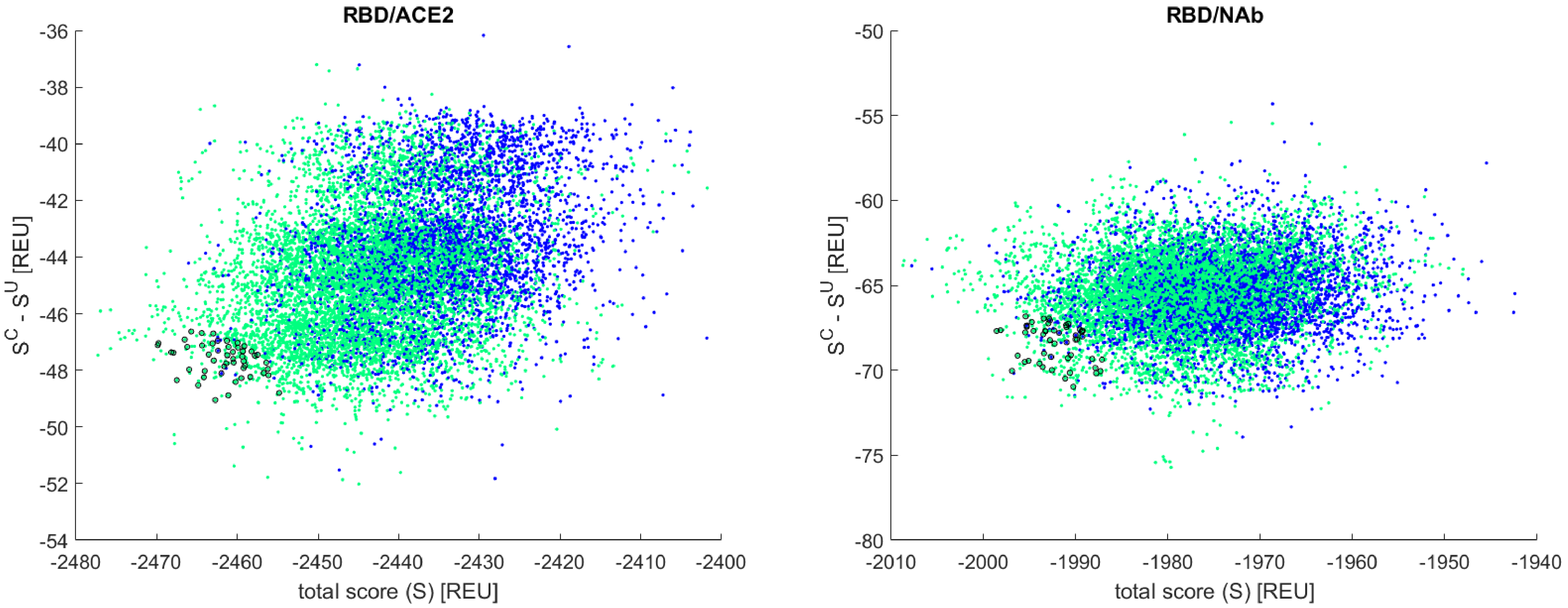
RBD/ACE2
-36
-38
-40
-42
-44
-46
-48
-50
-52
-54
$S^C - S^U$ [REU]
-2480
-2470
-2460
-2450
-2440
-2430
-2420
-2410
-2400
total score (S) [REU]
RBD/NAb
-50
-55
-60
-65
-70
-75
-80
$S^C - S^U$ [REU]
-2010
-2000
-1990
-1980
-1970
-1960
-1950
-1940
total score (S) [REU]

S6

Delta

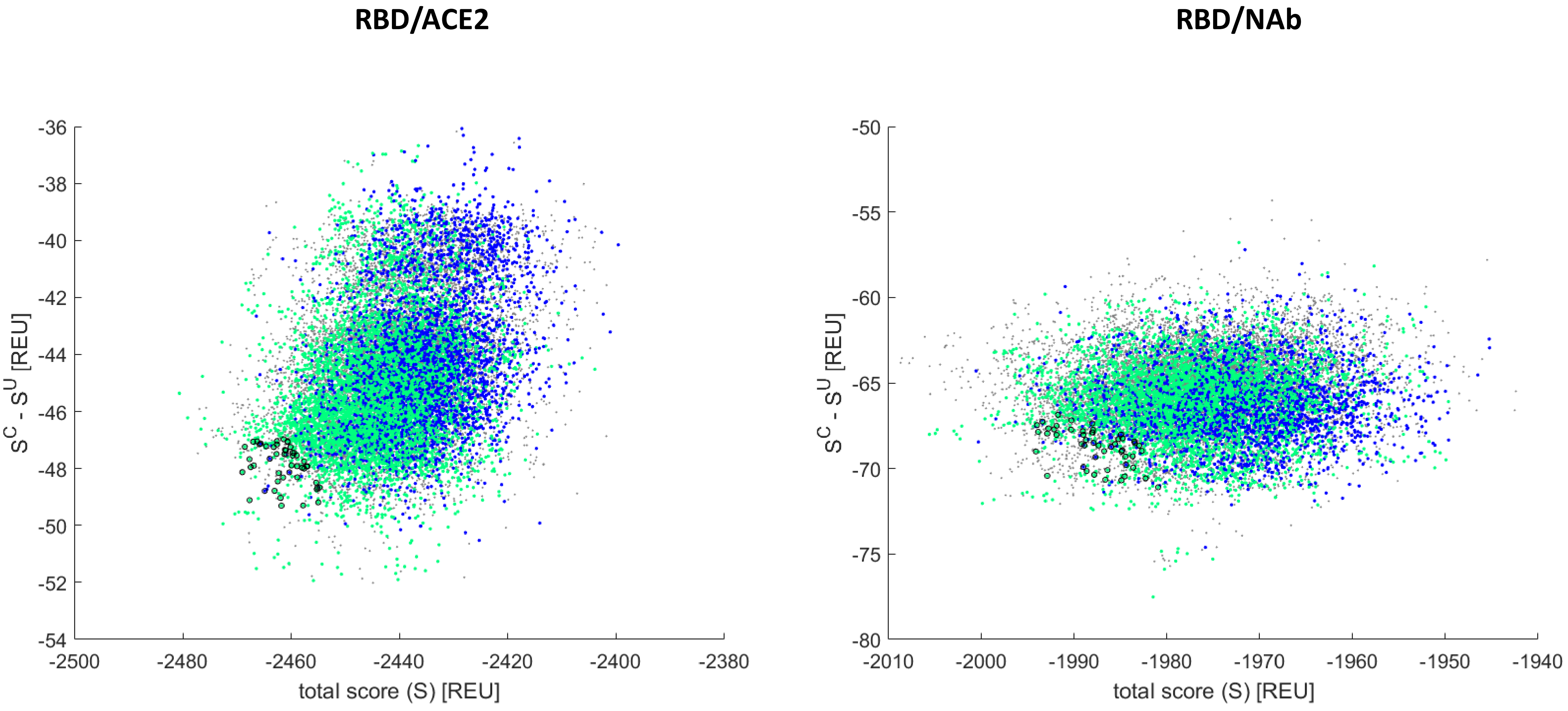
RBD/ACE2
RBD/NAb
-36
-38
-40
-42
-44
-46
-48
-50
-52
-54
S^C - S^U [REU]
-2500
-2480
-2460
-2440
-2420
-2400
-2380
total score (S) [REU]
-50
-55
-60
-65
-70
-75
-80
S^C - S^U [REU]
-2010
-2000
-1990
-1980
-1970
-1960
-1950
-1940
total score (S) [REU]

S7

Gamma

RBD/ACE2

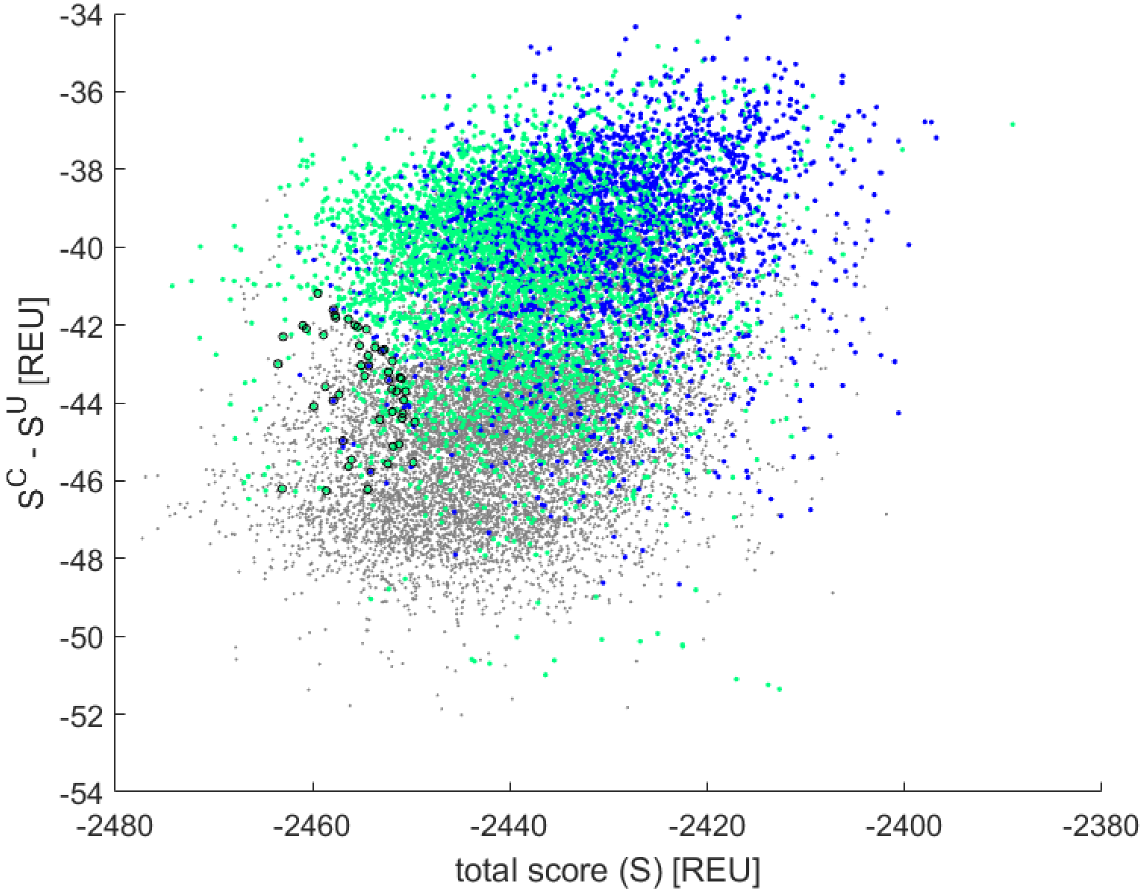

RBD/NAb

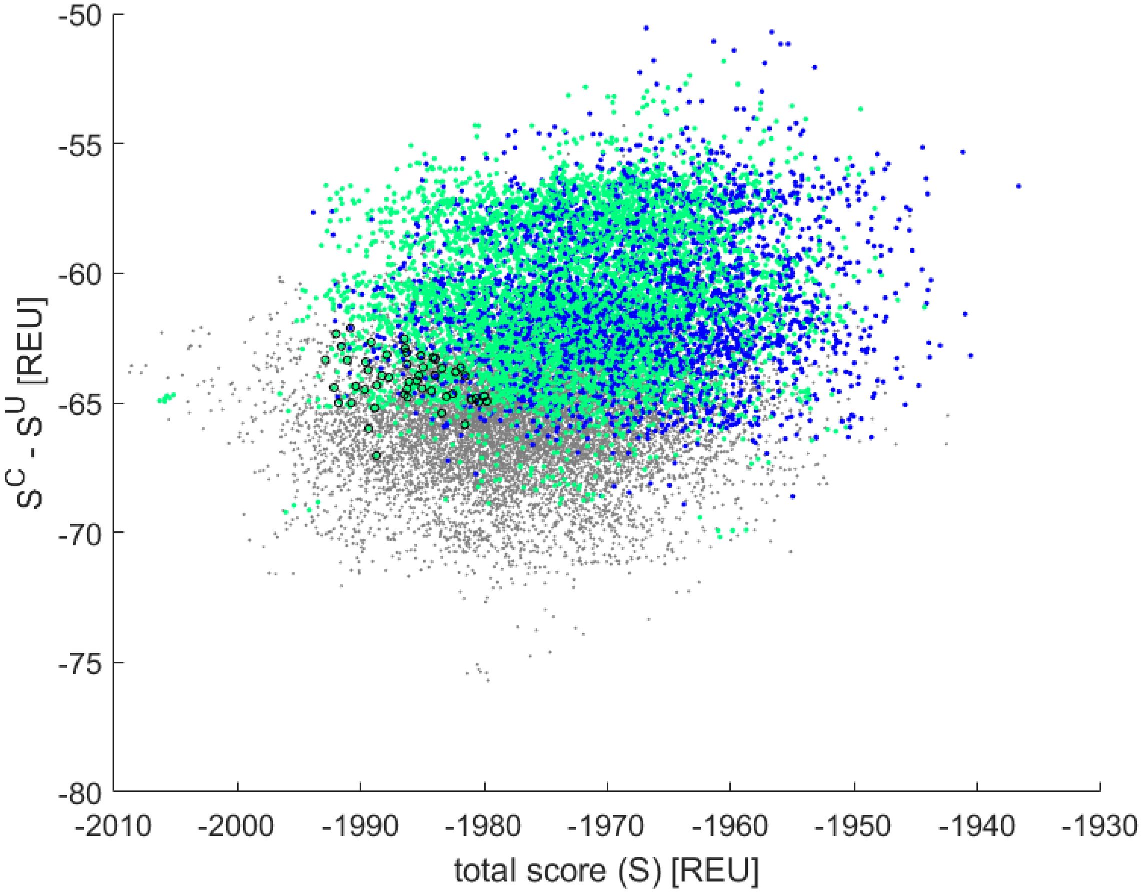

S8

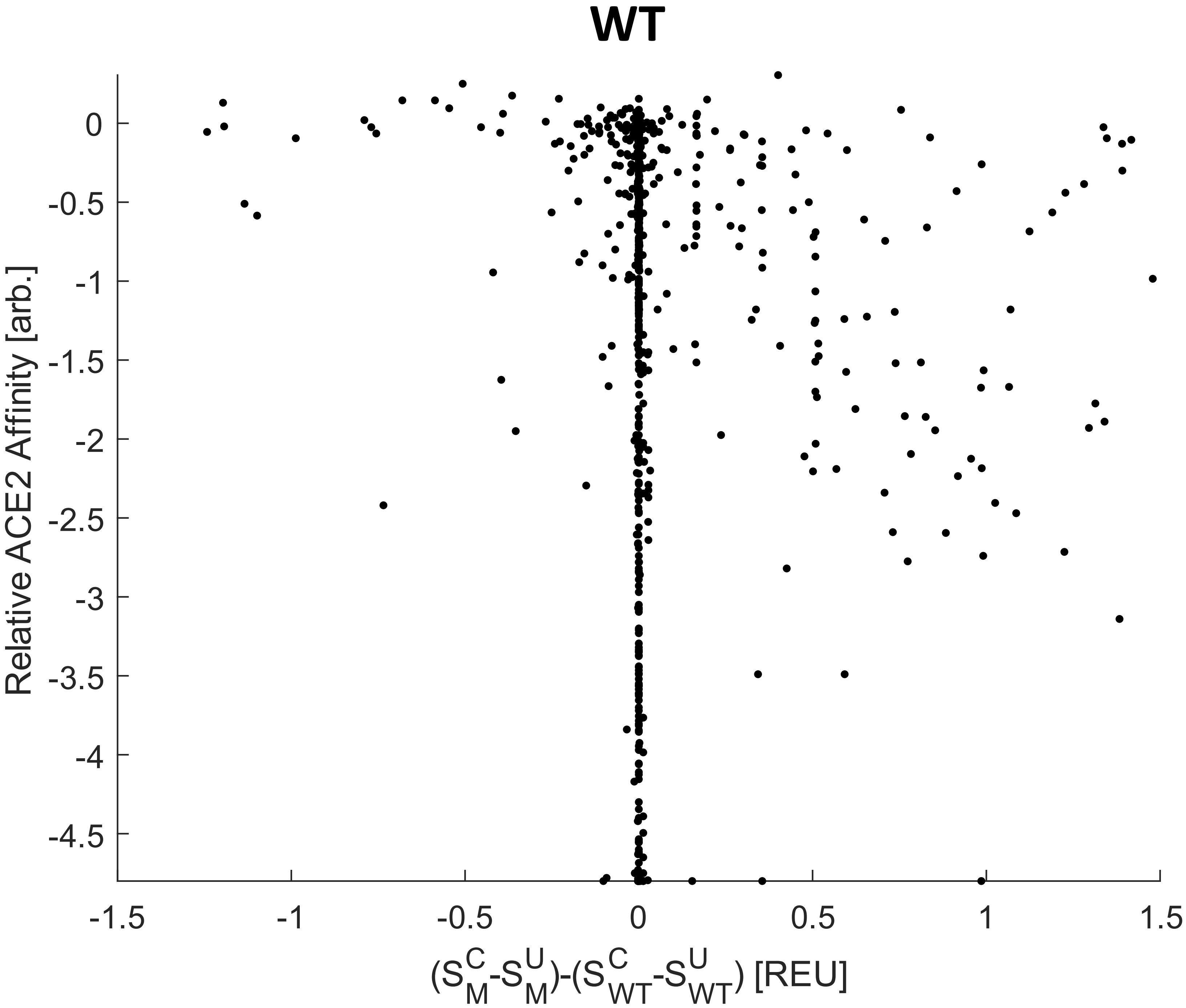
WT
Relative ACE2 Affinity [arb.]
0
-0.5
-1
-1.5
-2
-2.5
-3
-3.5
-4
-4.5
-1.5
-1
-0.5
0
0.5
1
1.5
$(S_M^C-S_M^U)-(S_{WT}^C-S_{WT}^U)$ [REU]

S9

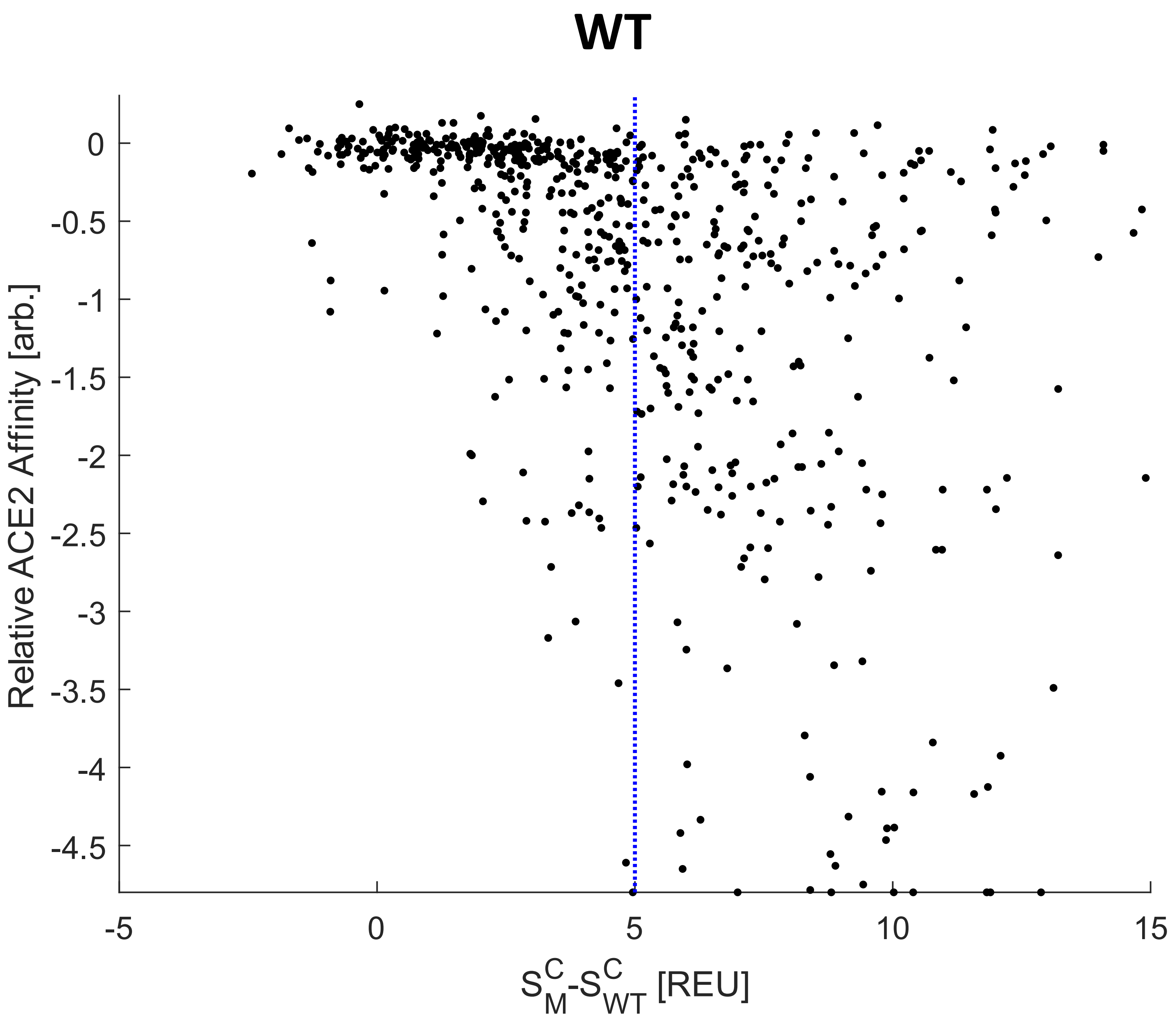
WT
Relative ACE2 Affinity [arb.]
0
-0.5
-1
-1.5
-2
-2.5
-3
-3.5
-4
-4.5
-5
0
5
10
15
S^C_M-S^C_{WT} [REU]

S10

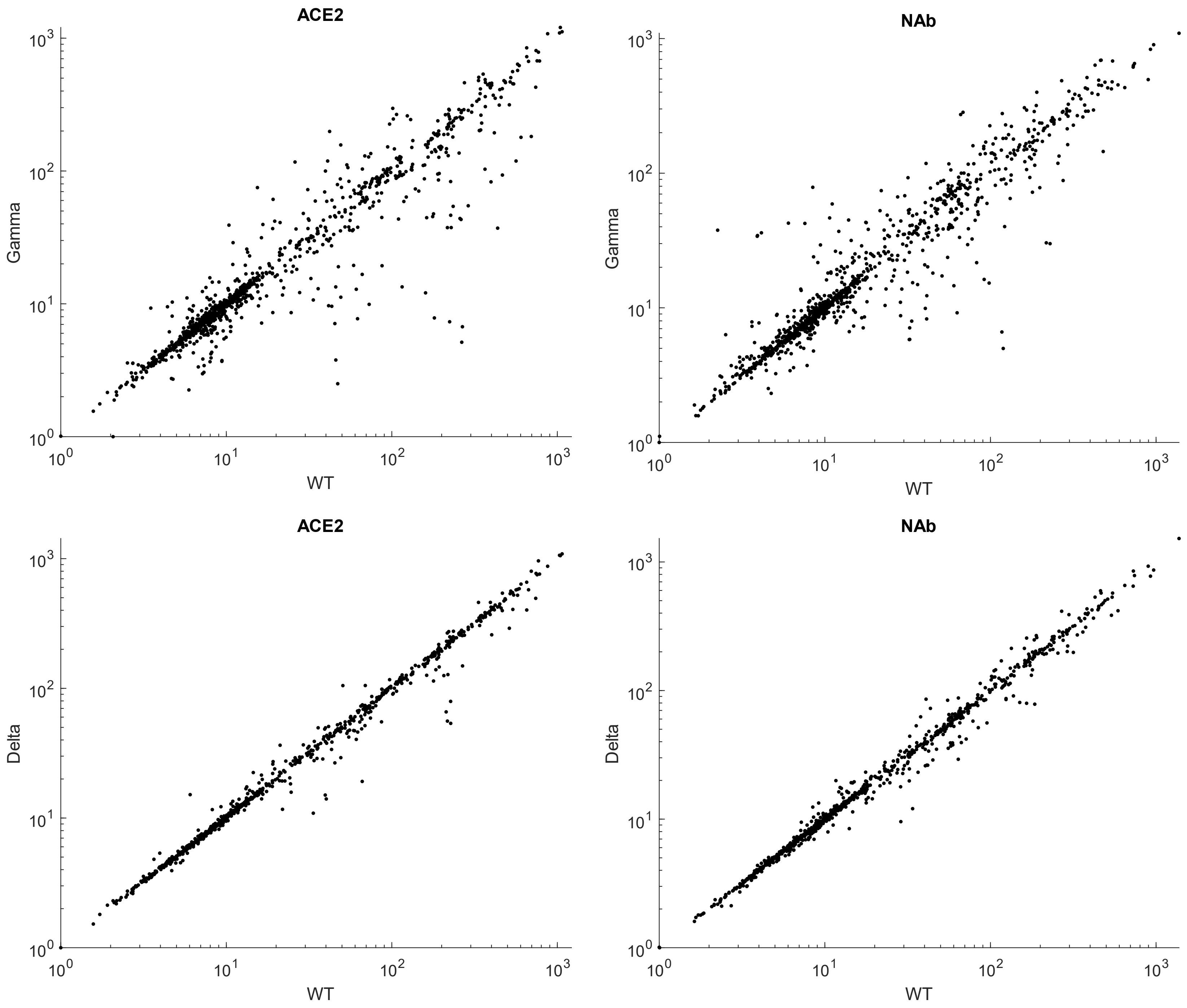
ACE2
NAb
ACE2
NAb
Gamma
Gamma
Delta
Delta
WT
WT
WT
WT
10^0
10^1
10^2
10^3

S11

Delta

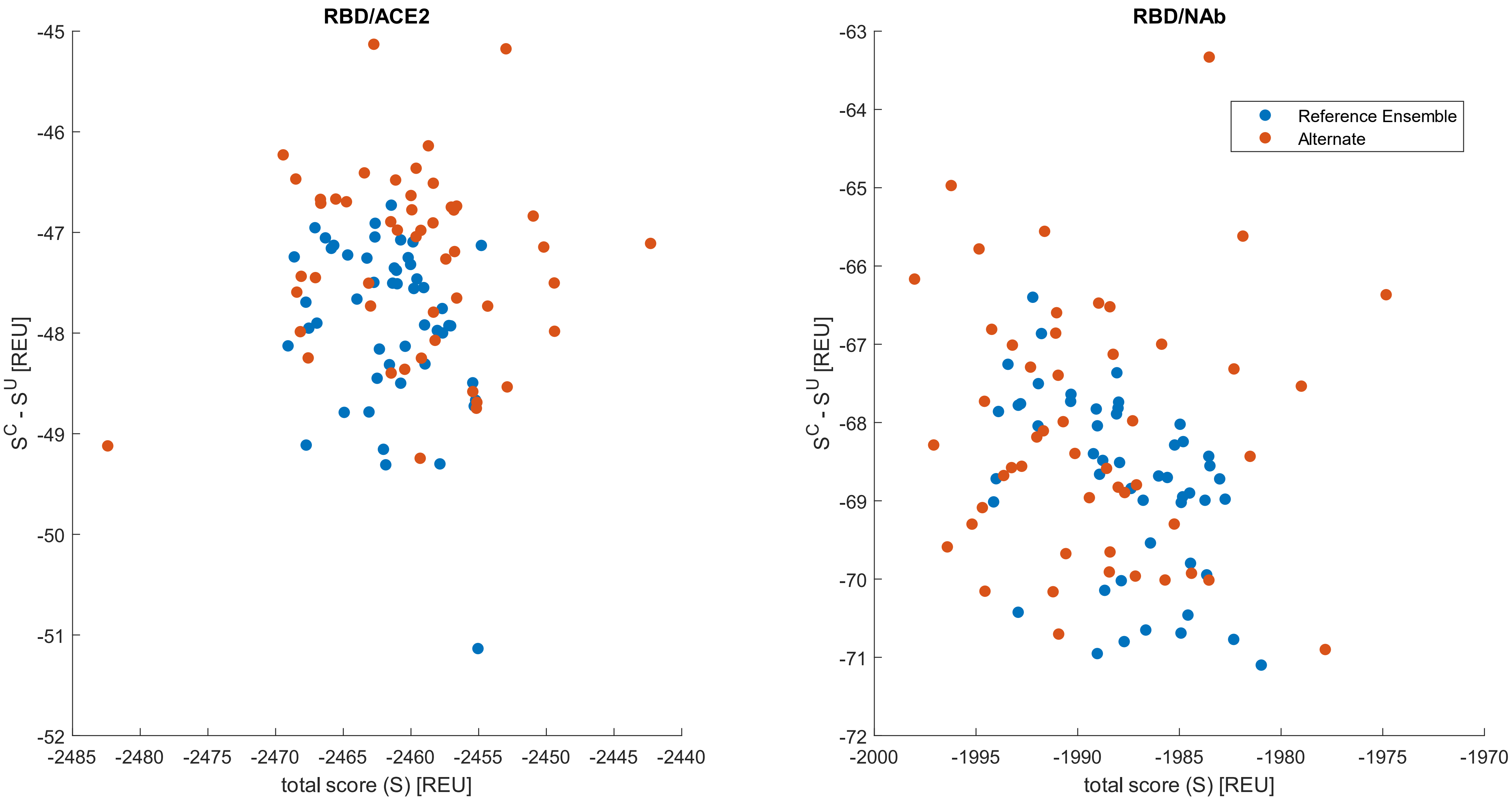
RBD/ACE2
-45
-46
-47
-48
-49
-50
-51
-52
S^C - S^U [REU]
-2485
-2480
-2475
-2470
-2465
-2460
-2455
-2450
-2445
-2440
total score (S) [REU]
RBD/NAb
-63
-64
-65
-66
-67
-68
-69
-70
-71
-72
-2000
-1995
-1990
-1985
-1980
-1975
-1970
Reference Ensemble
Alternate

S12

Gamma

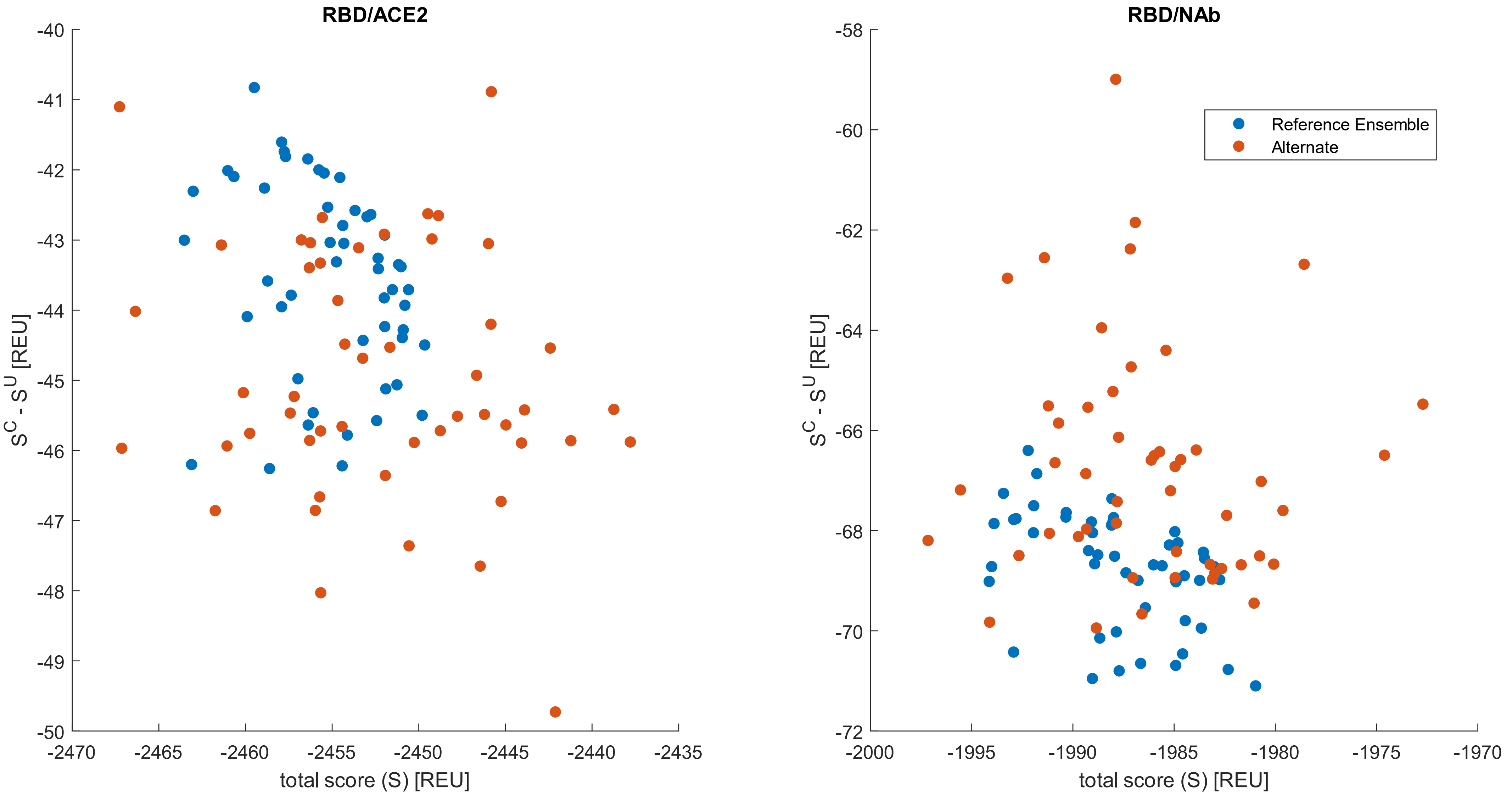
RBD/ACE2
RBD/NAb
Reference Ensemble
Alternate
$S^C - S^U$ [REU]
total score (S) [REU]
-40
-41
-42
-43
-44
-45
-46
-47
-48
-49
-50
-2470
-2465
-2460
-2455
-2450
-2445
-2440
-2435
-58
-60
-62
-64
-66
-68
-70
-72
-2000
-1995
-1990
-1985
-1980
-1975
-1970

Table S1

1) fastRelaxSpecRep.xml, which includes backbone minimization and sidechain repacking, is conducted using the following .xml file. This is applied to the WT crystal structure; to the crystal structure after the variant mutations have been introduced; and to the WT conformational ensemble after the variant mutations have been introduced to generate the alternate variant conformations. 5 repetitions are conducted in the below example command line.

Example Command Line:

```
.../rosetta_scripts.static.linuxgccrelease -parser:protocol .../fastRelaxSpecRep.xml -parser:script_vars numRep=5 -out:suffix suffix -out:path:pdb .../outFolder -out:path:score /dev/null -s .../input.pdb
```

____

```
<ROSETTASCRIPTS>
	<SCOREFXNS>
	</SCOREFXNS>
	<FILTERS>
	</FILTERS>
	<TASKOPERATIONS>
	</TASKOPERATIONS>
	<MOVERS>
		This mover does a fast relaxation. The input complex is should be pre-relaxed (prior to mutation).
		<FastRelax name="relax" disable_design="1" repeats="%%numRep%%"/>
	</MOVERS>
	<PROTOCOLS>
		Fast-relax the mutated complex.
		<Add mover_name="relax"/>
	</PROTOCOLS>
```

```
</ROSETTASCRIPTS>
```

---

2) Example resfile which is used to introduce mutations without repacking other residues. This example modifies site 403 on chain E to be Alanine(A).

```
NATRO
USE_INPUT_SC
start

403 E PIKAA A EX 1 EX 2 EX 3 EX 4
```

---

3) mutAndPack.xml, which introduces a mutation specified by the resfile. Note that (as in the above example)

if the resfile does not specify sidechain repacking, only the mutation is introduced with the remaining residues unchanged.

Example Command Line:

```
.../rosetta_scripts.static.linuxgccrelease -parser:protocol .../mutAndPack.xml -parser:script_vars resfile_path=.../resfile.dat -out:suffix suffix -out:path:pdb .../outFolder -out:path:score /dev/null -s input.pdb
```

```
<ROSETTASCRIPTS>
	<SCOREFXNS>
	</SCOREFXNS>
```

<FILTERS>

</FILTERS>

<TASKOPERATIONS>

This task operation is used to specify the location of the resfile to use for design. Note this can also be used to specify "dummy design" X->X

<ReadResfile name="resfile" filename="%%resfile_path%%"/>

</TASKOPERATIONS>

<MOVERS>

This mover packs rotamers with design as specified in the resfile named generic.resfile per the above.

<PackRotamersMover name="mut_and_pack" task_operations="resfile"/>

</MOVERS>

<PROTOCOLS>

First mutate the complex

<Add mover_name="mut_and_pack"/>

</PROTOCOLS>

</ROSETTASCRIPTS>

---

4) runInterfaceDDGandRMSD.xml which may be used to calculate the interface energy; identify interface residues;

and calculate the interface RMSD relative to a reference structure over a comma separated list of residues.

Example Command Line:

.../rosetta_scripts.static.linuxgccrelease -parser:protocol .../runInterfaceDDGandRMSD.xml -parser:script_vars pSep=1 chain1=A chain2=E interString=comma_separated_list_of_interface_residues pIn=0 interface_ID=A_E pUnbound=1 pBound=0 rUnbound=0 rBound=0 chainstomove=E -in:file:native .../reference.pdb -out:file:score_only .../score.txt -s input.pdb

---

```
<ROSETTASCRIPTS>
	<SCOREFXNS>
		<ScoreFunction name="standardfxn" weights="ref2015"/>
	</SCOREFXNS>
	<RESIDUE_SELECTORS>
			<Chain name="chA" chains="%%chain1%%" />
			<Chain name="chB" chains="%%chain2%%" />
			InterfaceByVector name="interfaceAB" grp1_selector="chA" grp2_selector="chB"
			<Index name="interfaceAB" resnums="%%interString%%"
error_on_out_of_bounds_index="1" reverse="0" />
	</RESIDUE_SELECTORS>
	<MOVE_MAP_FACTORIES>
			<MoveMapFactory name="Interface">
	<Backbone residue_selector="interfaceAB"/>
	<Chi residue_selector="interfaceAB"/>
			</MoveMapFactory>
	</MOVE_MAP_FACTORIES>
	<SIMPLE_METRICS>
			 SelectedResidueCountMetric name="nres_int" residue_selector="interfaceAB"
			<RMSDMetric name="Irmsd" rmsd_type="rmsd_protein_bb_ca"
residue_selector="interfaceAB" use_native="1" super="1"/>
			<RMSDMetric name="chB_rmsd" rmsd_type="rmsd_protein_bb_ca"
residue_selector="chB" use_native="1" super="1"/>
			<RMSDMetric name="chA_rmsd" rmsd_type="rmsd_protein_bb_ca"
residue_selector="chA" use_native="1" super="1"/>
	</SIMPLE_METRICS>
	<MOVERS>
			This mover applies the total score
			<ScoreMover name="apply_score" scorefxn="standardfxn"/>
```

```
This mover calculates the interface energy.

<InterfaceAnalyzerMover name="calc_interface" pack_separated="%%pSep%%" pack_input="%%pIn%%" interface_sc="1" interface="%%interface_ID%%"/>

This mover does a fast relaxation. The input complex is should be pre-relaxed (prior to mutation).

<FastRelax name="relax" disable_design="1"/>

This mover calculates DDG

<ddG name="standard_ddG_mover" scorefxn="standardfxn" relax_mover="relax" repack_unbound="%%pUnbound%%" repack_bound="%%pBound%%" relax_bound="%%rBound%%" relax_unbound="%%rUnbound%%" translate_by="1000.0" chain_name="%%chainstomove%%"/>


<RunSimpleMetrics name="run_metrics1" metrics="Irmsd" prefix="I_"/>

<RunSimpleMetrics name="run_metrics2" metrics="chB_rmsd" prefix="chB_"/>

<RunSimpleMetrics name="run_metrics3" metrics="chA_rmsd" prefix="chA_"/>

</MOVERS>

<PROTOCOLS>

First get total score

<Add mover_name="apply_score"/>

Then do interface calculation

<Add mover_name="calc_interface"/>

Then calc ddg

<Add mover_name="standard_ddG_mover"/>


Finally calc RMSD

<Add mover_name="run_metrics1"/>

<Add mover_name="run_metrics2"/>

<Add mover_name="run_metrics3"/>

</PROTOCOLS>

</ROSETTASCRIPTS>
```

---

5)runInterfaceAndDDG.xml, alternative to above when RMSD calculation is not necessary.

Example Command Lines:

Returns Only Scores:

.../rosetta_scripts.static.linuxgccrelease -parser:protocol .../runInterfaceAndDDG.xml -parser:script_vars pSep=0 pIn=0 interface_ID=A_E pUnbound=0 pBound=0 rUnbound=0 rBound=0 chainstomove=E -out:file:score_only output.txt -s input.pdb

Returns Only PDBs with Interface Residues Specified:

.../rosetta_scripts.static.linuxgccrelease -parser:protocol ../rosetta/my_xml/runInterfaceAndDDG.xml -parser:script_vars pSep=0 pIn=0 interface_ID=HL_E pUnbound=0 pBound=0 rUnbound=0 rBound=0 chainstomove=E -out:path:pdb .../outFolder -out:path:score /dev/null -s input.pdb

---

```
<ROSETTASCRIPTS>
	<SCOREFXNS>
		<ScoreFunction name="standardfxn" weights="ref2015"/>
	</SCOREFXNS>
	<FILTERS>
	</FILTERS>
	<TASKOPERATIONS>
	</TASKOPERATIONS>
	<MOVERS>
		This mover applies the total score
		<ScoreMover name="apply_score" scorefxn="standardfxn"/>
```

This mover calculates the interface energy.

<InterfaceAnalyzerMover name="calc_interface" pack_separated="%%pSep%%" pack_input="%%pIn%%" interface_sc="1" interface="%%interface_ID%%"/>

This mover does a fast relaxation. The input complex is should be pre-relaxed (prior to mutation).

<FastRelax name="relax" disable_design="1"/>

This mover calculates DDG

<ddG name="standard_ddG_mover" scorefxn="standardfxn" relax_mover="relax" repack_unbound="%%pUnbound%%" repack_bound="%%pBound%%" relax_bound="%%rBound%%" relax_unbound="%%rUnbound%%" translate_by="1000.0" chain_name="%%chainstomove%%"/>

</MOVERS>

<PROTOCOLS>

First get total score

<Add mover_name="apply_score"/>

Then do interface calculation

<Add mover_name="calc_interface"/>

Then calc ddg

<Add mover_name="standard_ddG_mover"/>

</PROTOCOLS>

</ROSETTASCRIPTS>
